# Intermittent hypoxia enhances the expression of hypoxia inducible factor HIF1A through histone demethylation

**DOI:** 10.1016/j.jbc.2022.102536

**Published:** 2022-09-27

**Authors:** Chloe-Anne Martinez, Yannasittha Jiramongkol, Neha Bal, Imala Alwis, Polina E. Nedoboy, Melissa M.J. Farnham, Mark D. White, Peter A. Cistulli, Kristina M. Cook

**Affiliations:** 1 The University of Sydney, Faculty of Medicine and Health, Charles Perkins Centre, Sydney, New South Wales, Australia; 2 The University of Sydney, Faculty of Science, Charles Perkins Centre, Sydney, New South Wales, Australia; 3 The University of Sydney, School of Chemistry, Sydney, New South Wales, Australia; 4 Heart Research Institute, The University of Sydney, Sydney, New South Wales, Australia

**Keywords:** hypoxia, hypoxia inducible factor, histone demethylase, hydroxylase, transcription, KDM, cDNA, complementary DNA, CHX, cycloheximide, FBS, fetal bovine serum, OSA, obstructive sleep apnea, PDMS, polydimethylsiloxane, qPCR, quantitative PCR

## Abstract

The cellular response to hypoxia is regulated through enzymatic oxygen sensors, including the prolyl hydroxylases, which control degradation of the well-known hypoxia inducible factors (HIFs). Other enzymatic oxygen sensors have been recently identified, including members of the KDM histone demethylase family. Little is known about how different oxygen-sensing pathways interact and if this varies depending on the form of hypoxia, such as chronic or intermittent. In this study, we investigated how two proposed cellular oxygen-sensing systems, HIF-1 and KDM4A, KDM4B, and KDM4C, respond in cells exposed to rapid forms of intermittent hypoxia (minutes) and compared to chronic hypoxia (hours). We found that intermittent hypoxia increases HIF-1α protein through a pathway distinct from chronic hypoxia, involving the KDM4A, KDM4B, and KDM4C histone lysine demethylases. Intermittent hypoxia increases the quantity and activity of KDM4A, KDM4B, and KDM4C, resulting in a decrease in histone 3 lysine 9 (H3K9) trimethylation near the *HIF1A* locus. We demonstrate that this contrasts with chronic hypoxia, which decreases KDM4A, KDM4B, and KDM4C activity, leading to hypertrimethylation of H3K9 globally and at the *HIF1A* locus. Altogether, we found that demethylation of histones bound to the *HIF1A* gene in intermittent hypoxia increases *HIF1A* mRNA expression, which has the downstream effect of increasing overall HIF-1 activity and expression of HIF target genes. This study highlights how multiple oxygen-sensing pathways can interact to regulate and fine tune the cellular hypoxic response depending on the period and length of hypoxia.

Chronic hypoxia is common in solid tumors and is associated with treatment resistance and aggressive disease (1). Along with chronically hypoxic regions, solid tumors contain regions of intermittent hypoxia, which arise from fluctuations in perfusion and red blood cell flux (2). Systemic intermittent hypoxia also occurs in obstructive sleep apnea (OSA), a common disorder characterized by repetitive interruptions in breathing during sleep due to collapse of the upper airway. Like chronic hypoxia, perfusion-limited intermittent hypoxia and OSA-induced intermittent hypoxia have been shown to promote a more malignant tumor phenotype, despite the different range of time scales for the hypoxic periods (3, 4). While there is no precise definition of the time periods for chronic or intermittent hypoxia, chronic hypoxia is generally defined as sustained low oxygen lasting for hours, while intermittent or cyclical hypoxia involves intermittent periods of low oxygen varying from minutes to hours/days punctuated by periods of increased oxygenation (2).

The cellular response to chronic hypoxia is largely coordinated by hypoxia inducible factors (HIFs). HIF is controlled by the prolyl hydroxylase (PHD) oxygen-sensing enzymes, which use oxygen to hydroxylate HIF-α subunits, targeting them for degradation. Factor inhibiting HIF (FIH) also uses oxygen to hydroxylate HIF-α subunits, which blocks binding to the transcriptional coactivators p300/CBP. In the presence of insufficient oxygen, PHD and FIH activity substantially decreases, enabling HIF-α to bind to HIF-β and initiate the transcription of target genes (5). Similar to chronic hypoxia, longer periods of intermittent hypoxia (hours to days) have been shown to increase HIF-1 activity (3). HIF-1α also increases in the carotid body in response to rapid intermittent hypoxia (minutes) using sleep apnea models through activation of mTOR and impaired prolyl hydroxylation. In contrast, HIF-2α decreases in the carotid body in rapid intermittent hypoxia through increased degradation by Ca^2+^-activated calpain proteases (6).

Epigenetic modifications of histones are also involved in the response to chronic hypoxia (7). In the nucleus of cells, DNA is wrapped around octamers of histone proteins and organized into chromatin. Methylation of histone tail lysine residues alters the accessibility of chromatin to transcription factors and therefore the expression of genes (8). Histone lysines can be monomethylated, dimethylated, or trimethylated, and these modifications can be gene activating or repressive depending on the location of the modified amino acid residue and the degree of methylation (7). Histone methylation levels are a balance between methylation by histone methyltransferases, known as KMTs, and demethylation by histone lysine demethylases known as KDMs (or JmjCs). KDMs belong to the same family of the 2-oxoglutarate (2OG)–dependent oxygenases as the PHDs and they require 2OG, oxygen, and Fe(II) to maintain their demethylase activity (7). Specific members of the KDM family have been found to act as enzymatic oxygen sensors, including KDM4A (9), KDM5A (10), and KDM6A (11), meaning that their activity is affected by changes in physiologically relevant oxygen levels. Chronic hypoxia inhibits KDM activity resulting in global hypermethylation of multiple histone lysine residues, altering the expression of several genes (10, 11). To our knowledge, no one has examined how KDMs behave in intermittent hypoxia or how KDMs interact with the HIF pathway under these conditions.

In this study, we describe a unique role for the KDM4A, KDM4B, and KDM4C histone demethylases in regulating the expression of *HIF1A* and therefore controlling the HIF-1 response in rapid intermittent hypoxia. This pathway is distinct from the canonical oxygen-dependent degradation pathway and does not occur in chronic hypoxia.

## Results

### Intermittent hypoxia increases HIF-1α protein and expression of known HIF-1 target genes

**Figure 1:**
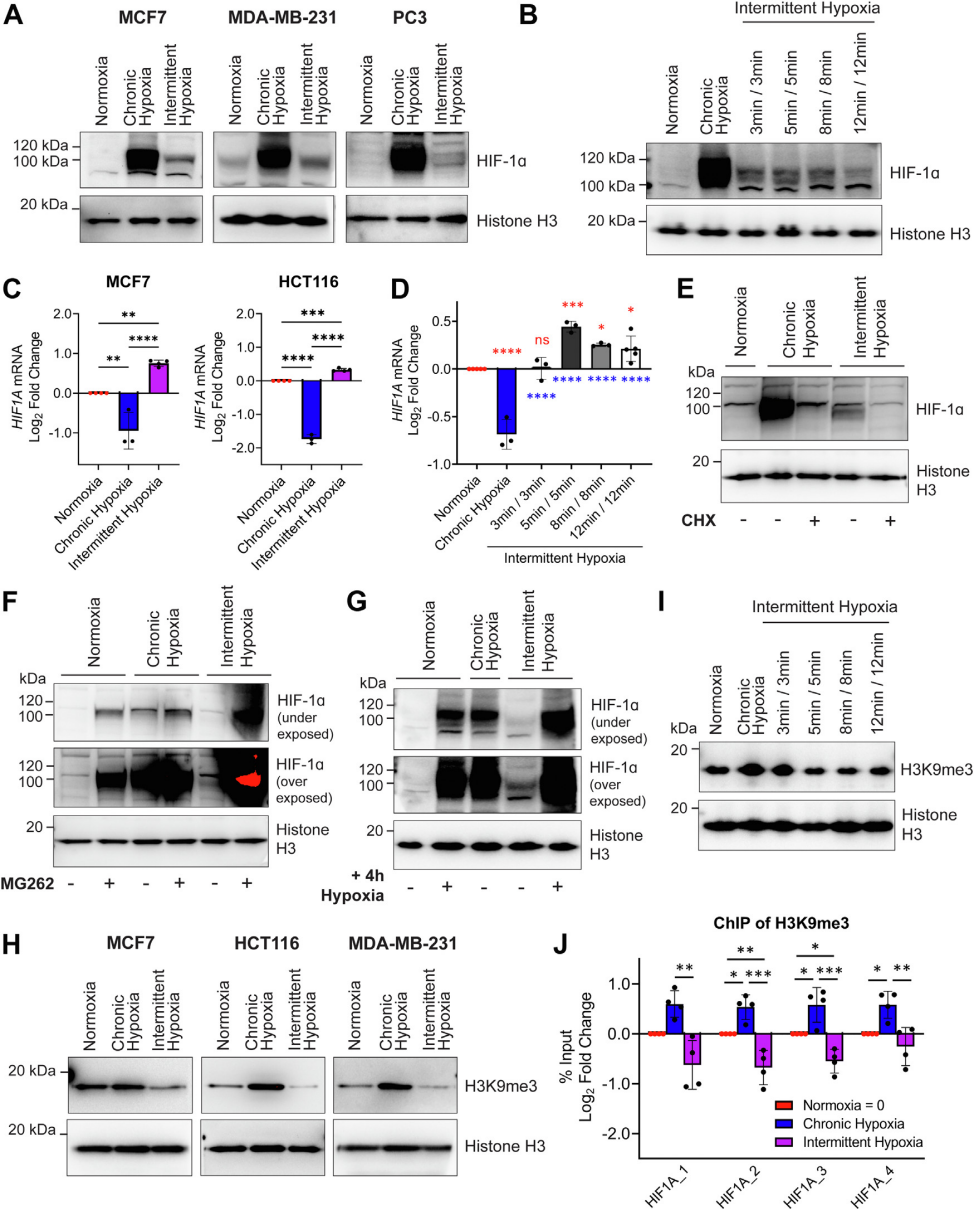
**Intermittent hypoxia increases *HIF1A* expression, decreases global H3K9me3, and decreases H3K9me3 at the *HIF1A* gene locus.***A*, nuclear HIF-1α levels in MCF7, MDA-MB-231, and PC3 cells exposed to normoxia, chronic hypoxia, and intermittent hypoxia (5 min/5 min). *B*, nuclear HIF-1α levels in MCF7 cells exposed to normoxia, chronic hypoxia, and varying periods of intermittent hypoxia. *C*, *HIF1A* mRNA levels in MCF7 and HCT116 cells exposed to normoxia, chronic hypoxia, and intermittent hypoxia (5 min/5 min). *D*, *HIF1A* mRNA levels in MCF7 cells exposed to normoxia, chronic hypoxia, and varying periods of intermittent hypoxia. Mean ± S.D. of n ≥ 3 experiments. Values are normalized to normoxia (Log2 scale). *E*, nuclear HIF-1α in HCT116 cells exposed to normoxia, chronic hypoxia, and intermittent hypoxia (5 min/5 min) treated with DMSO or cycloheximide (CHX). *F*, nuclear HIF-1α in HCT116 cells exposed to normoxia, chronic hypoxia, and intermittent hypoxia (5 min/5 min) with DMSO or MG262. *G*, nuclear HIF-1α in MCF7 cells exposed to 18 h normoxia, chronic hypoxia, and intermittent hypoxia (5 min/5 min) with or without an additional 4 h of chronic hypoxia. *H*, H3K9me3 levels in HCT116, MCF7, and MDA-MB-231 cells exposed to normoxia, chronic hypoxia, and intermittent hypoxia (5 min/5 min). *I*, H3K9me3 levels in MCF7 cells exposed to normoxia, chronic hypoxia, and varying periods of intermittent hypoxia. Histone H3 is used as a loading control for (*A*, *B* and *E*). *J*, chromatin immunoprecipitation was performed in MCF7 cells for H3K9me3 and enrichment on four different sites along the *HIF1A* gene relative to the total amount of input chromatin was quantified by qPCR. Values are normalized to normoxia (Log2 scale). Mean ± SD of n = 4. ∗*p* < 0.05, ∗∗*p* < 0.01, ∗∗∗*p* < 0.001, ∗∗∗∗*p* < 0.0001. *Asterisks* above a line compare data points connected by the line. *Red asterisks* above the data compare that condition to normoxia. *Blue asterisks* below the data compare that condition to chronic hypoxia. DMSO, dimethyl sulfoxide; qPCR, quantitative PCR.

We have previously shown that HIF-1α protein and HIF-1 target gene expression increases in intermittent hypoxia (5 min/5 min cycles) in HCT116 cells (12). To examine if this is a generalized cellular response, we exposed MCF7, MDA-MB-231, and PC3 cells to 18 h of intermittent hypoxia (alternating 5 min/5 min cycles of normoxia/hypoxia (see Fig. S8 for pericellular oxygen measurements) and compared the results to 18 h of normoxia (12% O_2_ v/v) or chronic hypoxia (0.5% O_2_ v/v). Relative to normoxia, HIF-1α protein increased under both chronic and intermittent hypoxia but to a lesser extent in intermittent hypoxia, similar to HCT116 cells (12) (Fig. 1*A*). We also found that expression of select HIF-1 target genes increased (Fig. S1). Overall, the increase in HIF-1α and HIF target gene expression in chronic and intermittent hypoxia supports the idea that HIF-1 is activated as a general response to both forms of hypoxia, and as we showed previously, the degree of gene expression is related to a hypoxia dose-dependent effect (12).

To assess how different protocols of intermittent hypoxia affect HIF-1α protein levels, we tested various lengths of normoxia, followed by an identical length of hypoxia, which was cycled for 18 h and compared to 18 h of normoxia or chronic hypoxia (see Experimental procedures for further details). As shown in Figure 1*B*, all tested periods of intermittent hypoxia (3 min/3 min, 5 min/5 min, 8 min/8 min, and 12 min/12 min, see Fig. S8 for pericellular oxygen measurements) increased HIF-1α protein to an intermediate level between normoxia and chronic hypoxia. This indicates that various short periods of intermittent hypoxia result in an increase in HIF-1α.

### Intermittent hypoxia increases *HIF1A* mRNA

Expression of the *HIF1A* gene is believed to be constitutive under most conditions (13), though exposure to chronic hypoxia dampens *HIF1A* expression (14). Having identified that HIF-1 activity increases in a wide range of cells exposed to intermittent hypoxia, we wanted to know if *HIF1A* mRNA expression was affected by intermittent hypoxia or if regulation of HIF-1α protein levels was occurring primarily through the oxygen-dependent posttranslational modifications pathway as in chronic hypoxia.

We found that intermittent hypoxia increases *HIF1A* mRNA in HCT116 cells ((12) and Fig. 1*C*) and MCF7 cells (Fig. 1*C*) when compared to normoxia. In contrast, when cells are exposed to chronic hypoxia, expression of *HIF1A* mRNA decreases (Fig. 1*C*). We also compared the levels of *HIF1A* mRNA in brain (U251), prostate (PC3), and additional breast (MDA-MB-231) cancer cell lines following exposure to normoxia, chronic, and intermittent hypoxia (Fig. S2). All cell lines showed a similar decrease in *HIF1A* mRNA in chronic hypoxia and an increase in *HIF1A* mRNA following intermittent hypoxia relative to normoxia. We also measured *EPAS1* (HIF-2α) expression following intermittent and chronic hypoxia to determine if expression was regulated in a similar manner to *HIF1A* and found that it was not (Fig. S3).

To assess how different protocols of intermittent hypoxia affected *HIF1A* gene expression, we tested the same cycling periods of intermittent hypoxia as shown in Figure 1*B* and measured *HIF1A* expression in MCF7 cells following exposure. All intermittent hypoxia protocols (5 min/5 min, 8 min/8 min, and 12 min/12 min) significantly increased *HIF1A* expression when compared to normoxia and chronic hypoxia, except 3 min/3 min, which did not change from normoxia (Fig. 1*D*). This indicates that expression of *HIF1A* is controlled differently in chronic hypoxia and intermittent hypoxia and this effect can depend on the period of the intermittent hypoxic cycles. The 5 min/5 min cycles of intermittent hypoxia promoted the largest increase in *HIF1A* mRNA.

### Increased *HIF1A* mRNA contributes to HIF-1 activity in intermittent hypoxia

To determine whether the increase in HIF-1α in intermittent hypoxia is dependent on the increase in *HIF1A* mRNA (Fig. 1*C*), HCT116 cells were treated with cycloheximide (CHX), a protein synthesis inhibitor, followed by either chronic or intermittent hypoxia for 6 h. CHX blocks *de novo* protein synthesis, therefore HIF-1α levels would reflect protein stabilization and degradation processes. As shown in Figure 1*E*, CHX reduced HIF-1α protein levels in both chronic and intermittent hypoxia, indicating that HIF-1α accumulation is dependent on continuous protein synthesis in both forms of hypoxia. This also suggests that HIF-1α still undergoes some proteasomal degradation in the presence of either form of hypoxia, though the rate of degradation under individual conditions may vary. Based on Figure 1*E*, the increase in HIF-1α observed in the absence of CHX for both chronic and intermittent hypoxia was due, at least in part, to new protein synthesis.

To further explore HIF-1α protein degradation in intermittent and chronic hypoxia, we treated HCT116 cells with MG262, a proteasome inhibitor followed by exposure to normoxia, chronic hypoxia, and intermittent hypoxia for 18 h. The proteasome is the primary degradation pathway for HIF-1α and by using MG262, we can observe how chronic and intermittent hypoxia affect HIF-1α protein expression without competing degradation processes affecting the final protein quantity. When normoxic HCT116 cells were treated with MG262 (Fig. 1*F*), HIF-1α accumulated due to blocked oxygen-dependent and oxygen-independent proteasomal degradation (lane 2). In chronic hypoxia, HIF-1α was stabilized (lane 3) and the addition of MG262 slightly increased HIF-1α protein (lane 4), confirming that low levels of proteasomal degradation still occurs in chronic hypoxia. In intermittent hypoxia, there were lower levels of HIF-1α protein (lane 5) when compared to chronic hypoxia (lane 3) but still more than in normoxia (lane 1, see overexposed blot, Fig. 1*F*). However, when MG262 is added to cells under intermittent hypoxia (lane 6), HIF-1α accumulated well above the observed level in chronic hypoxia, so much so that it was difficult to capture all conditions within the dynamic range of chemiluminescent detection. This indicates that more proteasomal degradation of HIF-1α occurs in intermittent hypoxia than in chronic hypoxia, which explains the lower HIF-1α levels in intermittent hypoxia relative to chronic hypoxia. Taken together with the *HIF1A* mRNA (Fig. 1, *C* and *D*) and CHX (Fig. 1*E*) data, this result also suggests that the increase in *HIF1A* mRNA expression in intermittent hypoxia directly contributes to the levels of HIF-1α protein, and when competing degradation processes are inhibited, this leads to a greater increase in HIF-1α (Fig. 1*F*).

These results led us to hypothesize that an increase in *HIF1A* mRNA synthesis in intermittent hypoxia (which does not occur in chronic hypoxia) enables a more robust HIF-1 response to a severe hypoxic event following intermittent hypoxia. To test this hypothesis, we exposed MCF7 cells to 18 h of normoxia, chronic hypoxia, or intermittent hypoxia that was immediately followed by an additional acute period of chronic hypoxia (4 h of chronic hypoxia) (total of 22 h). As expected, 18 h of normoxia showed no HIF-1α (Fig. 1*G*, lane 1). The addition of 4 h of chronic hypoxia following 18 h of normoxia led to a substantial increase in HIF-1α (lane 2), similar to the amount of HIF-1α present after 18 h of chronic hypoxia (lane 3). Eighteen hours of intermittent hypoxia led to a moderate increase in HIF-1α (see overexposed blot lane 4, Fig. 1*G*). However, the largest increase of HIF-1α in any of the conditions occurred when cells were exposed to 18 h of intermittent hypoxia followed by 4 h of chronic hypoxia (lane 5). Similar to the MG262 data, this experiment demonstrates that the increased *HIF1A* mRNA in intermittent hypoxia is translated into higher levels of HIF-1α protein, though in intermittent hypoxia some of this is likely degraded *via* oxygen-dependent degradation. When oxygen-dependent degradation is halted following intermittent hypoxia exposure, *via* a brief exposure to more severe chronic hypoxia, there is potential for a more robust HIF-1α response due to the increased level of *HIF1A* expression and protein synthesis generated by the preceding intermittent hypoxia.

### Histone 3 lysine 9 trimethylation (H3K9me3) increases in chronic hypoxia while decreasing in intermittent hypoxia

We identified that intermittent hypoxia increases *HIF1A* mRNA expression, while chronic hypoxia dampens *HIF1A* mRNA expression, indicating differential regulation depending on the type of hypoxic exposure. Histone methylation patterns alter the expression of genes and chronic hypoxia is associated with histone hypermethylation (7, 10, 11), while the effect of intermittent hypoxia on histone methylation has not been investigated. Furthermore, the methylation status of histone 3 lysine 9 (H3K9) has been previously linked to expression of the *HIF1A* gene (15), therefore we measured H3K9 trimethylation (H3K9me3) following exposure to normoxia, chronic hypoxia, and intermittent hypoxia. In agreement with previous studies (10, 15), we found that H3K9me3 increases in MCF7, HCT116, and MDA-MB-231 cells exposed to chronic hypoxia relative to normoxia (Fig. 1*H*). Surprisingly, and in contrast to chronic hypoxia, we found that intermittent hypoxia decreased levels of H3K9me3 below the levels observed in normoxia (Fig. 1*H*). H3K9me3 is associated with heterochromatin and gene silencing; therefore a global decrease in H3K9me3 induced by intermittent hypoxia is likely resulting in an increase in expression of associated genes.

To determine how the observed H3K9me3 changes affect other H3K9 methylation states, we measured monomethylation and dimethylation of H3K9 in MCF7 cells following exposure to the 5 min/5 min intermittent hypoxia protocol, which yielded the greatest fold increase in *HIF1A* mRNA. We found that global H3K9me2 levels slightly decreased following intermittent hypoxia (Fig. S4), while global H3K9me1 levels remained the same.

To assess how varying periods of intermittent hypoxia affect H3K9me3, we measured trimethylation levels in MCF7 cells following varying lengths of alternating normoxia and hypoxia. We found that 5 min/5 min, 8 min/8 min, and 12 min/12 min intermittent hypoxia cycles all decreased global H3K9me3 as compared to chronic hypoxia (Fig. 1*I*). Interestingly, 3 min/3 min cycles did not decrease H3K9me3 (Fig. 1*I*) and this corresponds with no increase in *HIF1A* mRNA under the same conditions (Fig. 1*D*).

### H3K9me3 accumulates at the *HIF1A* gene in chronic hypoxia and decreases at the *HIF1A* gene in intermittent hypoxia

H3K9me3 is a repressive marker for *HIF1A* gene expression in chronic hypoxia (15). To determine whether the decrease in H3K9me3 in intermittent hypoxia (Fig. 1*H*) was specifically occurring at the *HIF1A* gene locus, we carried out a chromatin immunoprecipitation quantitative PCR (qPCR) assay using MCF7 cells. Following exposure to normoxia, chronic hypoxia, and intermittent hypoxia, cells were lysed and H3K9me3 was immunoprecipitated. Coimmunoprecipitated DNA was purified and then subjected to qPCR using primers designed to detect *HIF1A* introns. For all regions measured, we found an increased quantity of the *HIF1A* gene in chronic hypoxia compared to normoxia (Fig. 1*J*). This indicates enrichment of H3K9me3 at the *HIF1A* gene in chronic hypoxia and supports previous work done in RKO cells (15). In contrast, there was a decrease in immunoprecipitated *HIF1A* in intermittent hypoxia compared to normoxia (Fig. 1*J*), indicating a loss of H3K9me3 at the *HIF1A* gene.

### Histone demethylases KDM4B, and KDM4C increase under intermittent and chronic hypoxia

Trimethyl (me3) (and dimethyl, me2) marks can be removed from H3K9 by the KDM4 demethylase subfamily, therefore we focused our attention on this group of enzymes. KDM4 enzymes require oxygen to function and belong to the same 2-OG-dependent family of oxygenases as the PHDs (7). KDM4A, KDM4B, and KDM4C have a low affinity for oxygen with *K*_*m*_ values measuring in a similar range as the PHD oxygen sensors (9, 11, 16), and KDM4B and KDM4C are transcriptionally upregulated by HIF under hypoxia (17, 18, 19).

We hypothesized that KDM4A, KDM4B, and KDM4C would lose activity in chronic hypoxia (increasing H3K9me3), while retaining activity in intermittent hypoxia due to sufficient periodic oxygenation, enabling ongoing demethylation of H3K9 similar to normoxia. Furthermore, if intermittent hypoxia increases expression of the HIF-target genes, *KDM4B* and *KDM4C*, as was seen with other HIF-target genes in Fig. S1, then this could lead to further H3K9 demethylase activity, even beyond the levels seen in normoxia.

**Figure 2:**
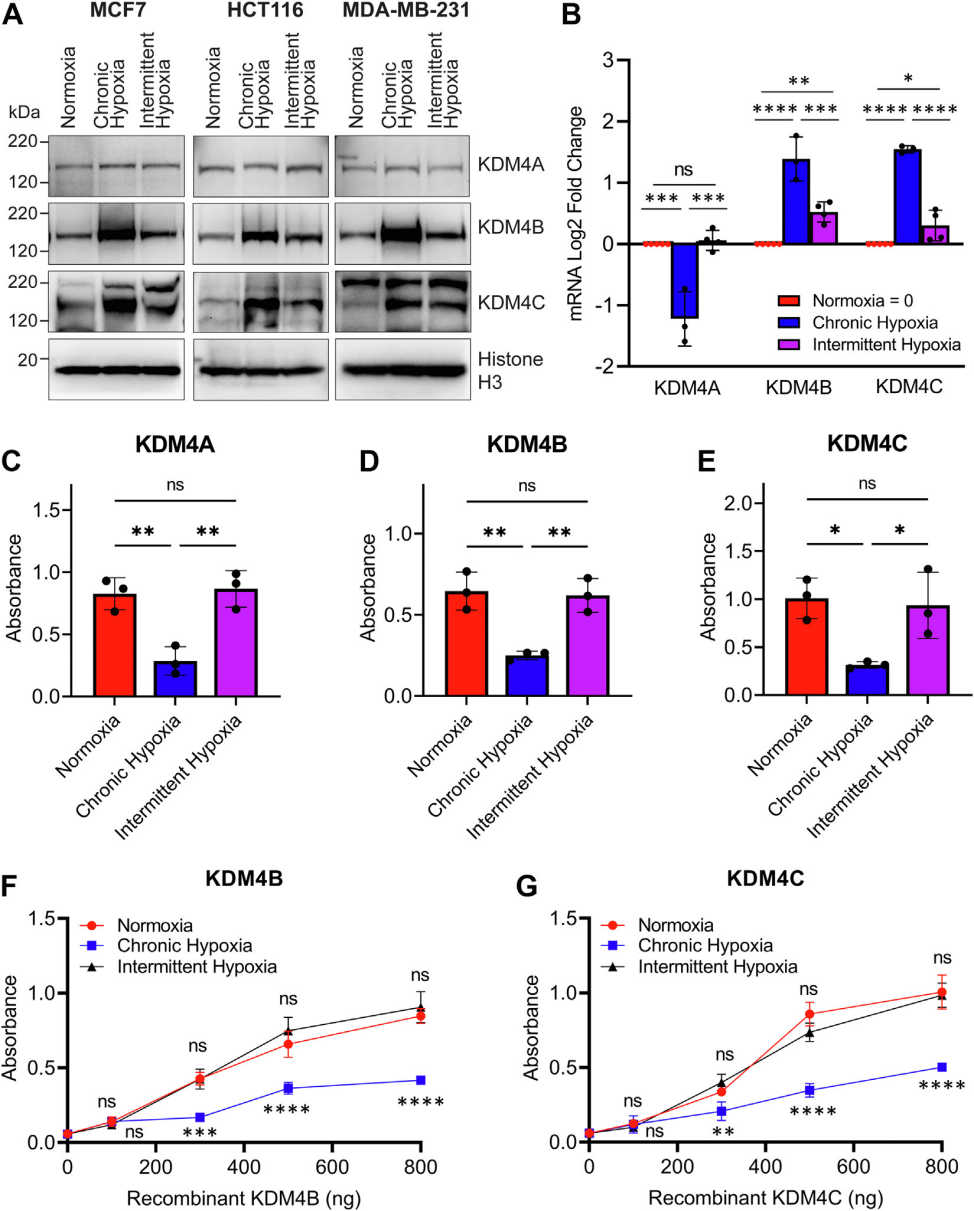
**Chronic hypoxia increases KDM4 expression but limits H3K9me3 demethylation while intermittent hypoxia increases KDM4 expression and enables H3K9me3 demethylation**. Cells were exposed to normoxia, chronic hypoxia, and intermittent hypoxia (5 min/5 min). *A*, KDM4A, KDM4B, and KDM4C in MCF7, HCT116, and MDA-MB-231 cells. Histone H3 is used as a loading control. *B*, mRNA levels of KDM4A, KDM4B, and KDM4C in HCT116. 500 ng of (*C*) KDM4A, (*D*) KDM4B, and (*E*) KDM4C recombinant proteins were added to microplate wells stably coated with H3K9me3 substrate and exposed to normoxia, chronic hypoxia, or intermittent hypoxia for 4 h. The relative amounts of demethylated H3K9me3 were determined using an HRP-linked antibody (mean ± SD n = 3). *F* and *G*, the same enzyme assay was conducted using a range of (*F*) KDM4B and (*G*) KDM4C enzyme concentrations. Results are the mean ± SD n = 2. ns = not significant, ∗*p* < 0.05, ∗∗*p* < 0.01, ∗∗∗*p* < 0.001, ∗∗∗∗*p* < 0.0001. *Asterisks* above the *red dots* compare normoxia to intermittent hypoxia. *Asterisks* below the *blue* square compare chronic hypoxia to intermittent hypoxia. Complete statistical analysis is presented in Table S1. HRP, horseradish peroxidase.

To test this hypothesis, we exposed MCF7, HCT116, and MDA-MB-231 cells to normoxia, chronic hypoxia, and intermittent hypoxia and assessed the expression of KDM4A, KDM4B, and KDM4C protein and mRNA. We found no changes in the protein levels of KDM4A in cells exposed to normoxia, chronic hypoxia, or intermittent hypoxia (Fig. 2*A*). In contrast, protein levels of KDM4B and KDM4C rose substantially in chronic hypoxia and to an intermediate level in intermittent hypoxia (Fig. 2*A*). *KDM4A* mRNA levels decreased in chronic hypoxia and did not change from normoxia in intermittent hypoxia, supporting previous data indicating that *KDM4A* is not a HIF-target gene or induced by hypoxia (7) (Fig. 2*B*). Expression of *KDM4B* and *KDM4C* mRNA increased in a similar pattern to the results in Fig. S1, where the strongest increase in HIF-target gene expression occurred under chronic hypoxia and a moderate increase in HIF-target gene expression occurred under intermittent hypoxia (Fig. 2*B*). These data indicate that under chronic and intermittent hypoxia, there are increased protein levels of KDM4B and KDM4C enzymes, with stable levels of KDM4A.

To confirm that the increase in KDM4B and KDM4C in intermittent hypoxia is HIF-dependent, like it has been shown in chronic hypoxia (17, 18), we employed *HIF1A* siRNA to knock down HIF-1 activity in MCF7 cells. Knockdown with *HIF1A* siRNA significantly decreased mRNA expression of *HIF1A* in chronic and intermittent hypoxia, which led to a significant reduction in *KDM4B* and *KDM4C* but not *KDM4A* mRNA levels (Fig. S5).

### KDM4A, KDM4B, and KDM4C demethylase activity at H3K9 is maintained in intermittent hypoxia and decreases in chronic hypoxia

In both intermittent and chronic hypoxia, KDM4B and KDM4C levels increase, while KDM4A levels remain stable (Fig. 2*A*). While increasing enzyme quantity can lead to an overall increase in enzyme activity, activity also relies on the availability of substrates and cofactors. Molecular oxygen is required for KDM4A, KDM4B, and KDM4C activity, which is limited in chronic hypoxia while present in varying levels in intermittent hypoxia. To compare the effects of chronic and intermittent hypoxia on KDM4A, KDM4B, and KDM4C activity, we measured enzymatic turnover of recombinant KDM4A, KDM4B, or KDM4C under normoxia, chronic hypoxia, and intermittent hypoxia using a KDM4 enzyme assay kit (Abcam). Five hundred nanograms of recombinant KDM4A, KDM4B, and KDM4C were added to microplate wells stably coated with H3K9me3 and exposed to 4 h of normoxia, chronic hypoxia, and intermittent hypoxia. Following exposure, demethylated H3K9me3 was captured using the provided primary antibody, and an horseradish peroxidase–linked secondary antibody was used for absorbance detection. All three enzymes (KDM4A: Fig. 2*C*, KDM4B: Fig. 2*D*, and KDM4C: Fig. 2*E*) had decreased demethylase activity in chronic hypoxia as expected. However, in intermittent hypoxia, there was no difference in the amount of demethylated H3K9 product produced when compared to normoxia at equivalent amounts of enzyme (Fig. 2, *C*–*E*). This indicates that at least for our protocol of intermittent hypoxia, there is sufficient oxygen present for enzyme activity and that our conditions have no apparent difference on demethylation than in normoxia. These results imply that chronic hypoxia limits the amount of oxygen available to KDM4 enzymes, decreasing demethylase activity. In contrast, oxygen is not a limiting factor for KDM4A, KDM4B, or KDM4C activity in intermittent hypoxia.

While KDM4 enzymatic activity is similar in normoxia and intermittent hypoxia when equivalent amounts of enzyme are used (Fig. 2, *C*–*E*), results from Figure 2*A* indicate that there are higher protein levels of KDM4B and KDM4C in cells exposed to intermittent hypoxia. The quantity of KDM4B and KDM4C also increases in chronic hypoxia but limited oxygen availability appears to hinder their activity (Fig. 2, *C*–*E*). Given that oxygen is not a limiting factor for KDM4 activity in intermittent hypoxia, we hypothesized that the increase in the quantity of KDM4B and KDM4C in intermittent hypoxia would result in overall greater H3K9me3 demethylation than in normoxia or chronic hypoxia.

To test this, we measured the demethylase activity of recombinant KDM4B and KDM4C using a range of enzyme concentrations under intermittent hypoxia and compared activity levels to normoxia and chronic hypoxia. Similar to the results from Figure 2, *D* and *E*, KDM4B and KDM4C demethylase activity in intermittent hypoxia was the same as normoxia at all comparable concentrations (Fig. 2, *F* and *G*). In contrast, KDM4B and KDM4C demethylase activity was significantly reduced in chronic hypoxia when compared to the same enzyme concentration in normoxia or intermittent hypoxia.

Altogether, these data show that intermittent hypoxia both increases the quantity of KDM4 enzymes (specifically KDM4B and KDM4C) and does not limit substrate availability for KDM4 enzymes (KDM4A, KDM4B, and KDM4C) to function optimally. This contrasts with normoxia, which produces the lowest quantity of KDM4 enzymes, and chronic hypoxia, where oxygen availability is limited. The combined effect of an increase in the quantity of KDM4 enzymes and oxygen availability for enzymatic function in intermittent hypoxia is an overall greater increase in KDM4 demethylase activity relative normoxia or chronic hypoxia.

### Histone demethylases KDM4A, KDM4B, and KDM4C regulate global H3K9me3 levels and H3K9me3 at the *HIF1A* locus under intermittent hypoxia

We showed that intermittent hypoxia increases KDM4B and KDM4C proteins and retains demethylase enzymatic activity of KDM4A, KDM4B, and KDM4C, leading to the highest levels of demethylation in intermittent hypoxia. The next step was to determine whether the increase in KDM4 activity is responsible for the demethylation of H3K9 and subsequent increase in *HIF1A* gene expression in intermittent hypoxia.

**Figure 3:**
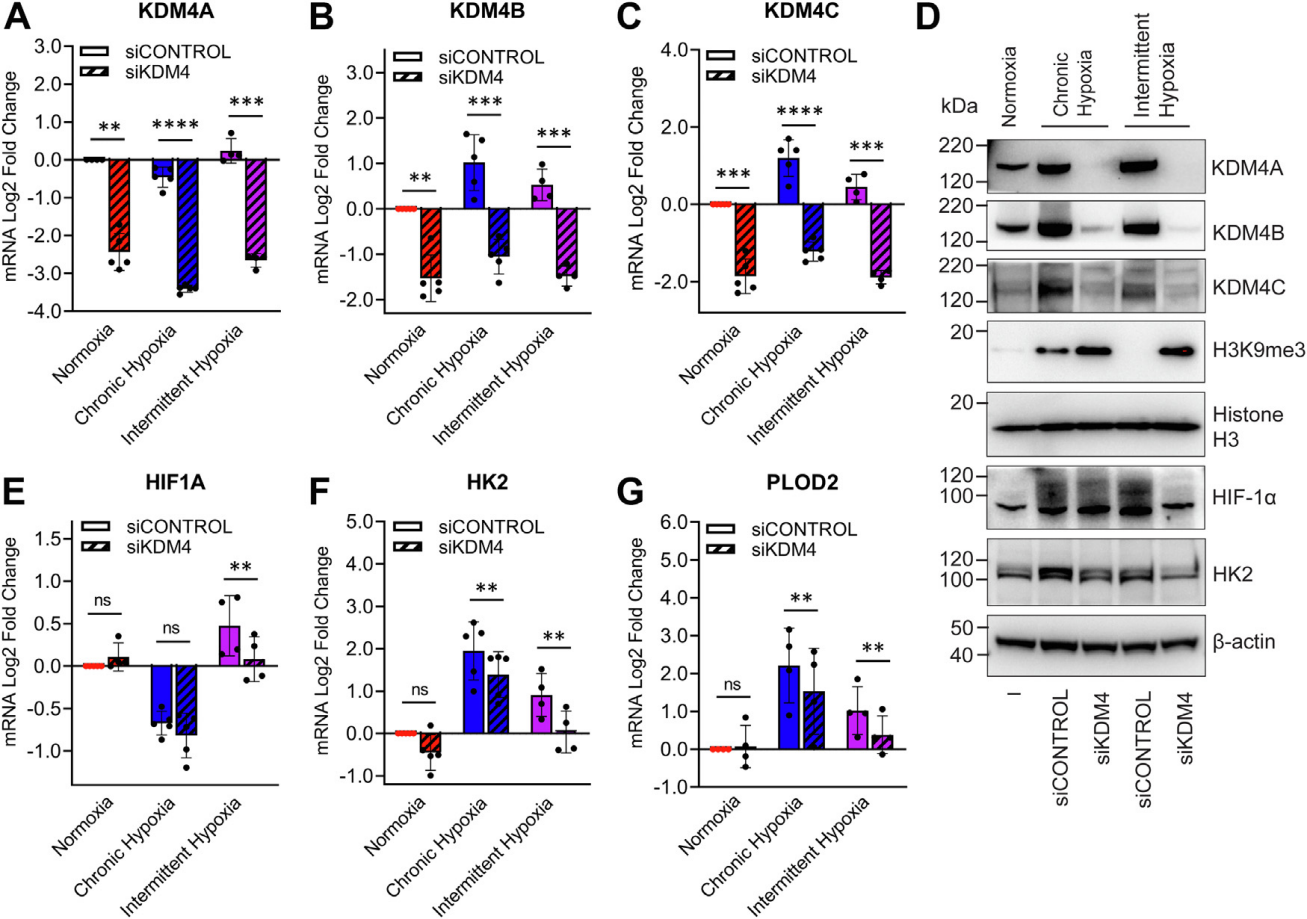
**Depletion of KDM4A/B/C increases trimethylation of H3K9 leading to a decrease in HIF-1α in intermittent hypoxia**. MCF7 cells were transfected with combined siKDM4A, siKDM4B, and siKDM4C (referred to as siKDM4) or siCONTROL followed by exposure to normoxia, chronic hypoxia (2% v/v), and intermittent hypoxia (5 min/5 min) over 18 h. mRNA expression of (*A*) KDM4A, (*B*) KDM4B, (*C*) KDM4C, (*E*) HIF1A, (*F*) HK2, and (*G*) PLOD2. All values are normalized to normoxia (Log2 scale). Results are the mean ± SD n ≥ 4 independent experiments. ns = not significant, ∗*p* < 0.05, ∗∗*p* < 0.01, ∗∗∗*p* < 0.001, ∗∗∗∗*p* < 0.0001. *D*, nuclear extracts of KDM4A, KDM4B, KDM4C, HIF-1α, H3K9me3, and histone H3 (loading control), and cytoplasmic extracts of HK2 and β-actin (loading control).

To test this, we used a combined siRNA approach to simultaneously knockdown KDM4A, KDM4B, and KDM4C in MCF7 cells. This was followed by measurement of H3K9me3 levels and *HIF1A* expression. Simultaneous knockdown of KDM4A, KDM4B, and KDM4C was highly effective at reducing mRNA and protein levels of each enzyme (Fig. 3, *A*–*D*). While H3K9me3 levels in chronic hypoxia were already high relative to normoxia, knocking down KDM4A, KDM4B, and KDM4C led to a greater increase in the levels of H3K9me3, indicating some residual demethylase activity in chronic hypoxia (Fig. 3*D*). Furthermore, where H3K9me3 was nearly undetectable in intermittent hypoxia, the triple knockdown increased H3K9me3 to similar levels to those seen in the chronic hypoxia triple knockdown, confirming that the methylation status of H3K9 in intermittent hypoxia is dependent on KDM4A-C activity (Fig. 3*D*). As shown in Figure 3*E*, the triple knockdown had little effect on *HIF1A* mRNA expression under normoxia and chronic hypoxia. However, in intermittent hypoxia, the triple knockdown significantly reduced *HIF1A* mRNA. The triple knockdown also reduced HIF-1α protein levels under intermittent hypoxia, while having little effect on HIF-1α in chronic hypoxia (2% oxygen v/v) (Fig. 3*D*). We also identified that knockdown of KDM4A, KDM4B, and KDM4C reduces the mRNA expression of known HIF-target genes, *HK2* and *PLOD2* (Fig. 3, *F* and *G*), and HK2 at the protein level (Fig. 3*D*). The knockdown data shown in Figure 3 confirm that the changes in H3K9me3 seen in intermittent hypoxia are due to KDM4A, KDM4B, and KDM4C.

### Peripheral intermittent hypoxia combined with spatially distinct core chronic hypoxia enhances overall HIF-1 activity in tumor spheroids

OSA is associated with higher rates of cancer and cancer mortality (3). The rapid intermittent hypoxia fluctuations associated with OSA have been hypothesized to alter tumor biology, possibly through enhanced activation of HIF-1. *In vitro* tumor spheroids replicate some aspects of *in vivo* tumor biology, and they contain both oxygenated, proliferating cells on the exterior and chronically hypoxic cells in the center (20). Therefore, we used a spheroid model to explore how chronic hypoxia present in the avascular tumor center might interact with OSA-driven intermittent hypoxia present in the blood stream, which would be experienced by cells on the tumor periphery. We first used this model to observe localized and total HIF activation under varying forms of hypoxia.

**Figure 4:**
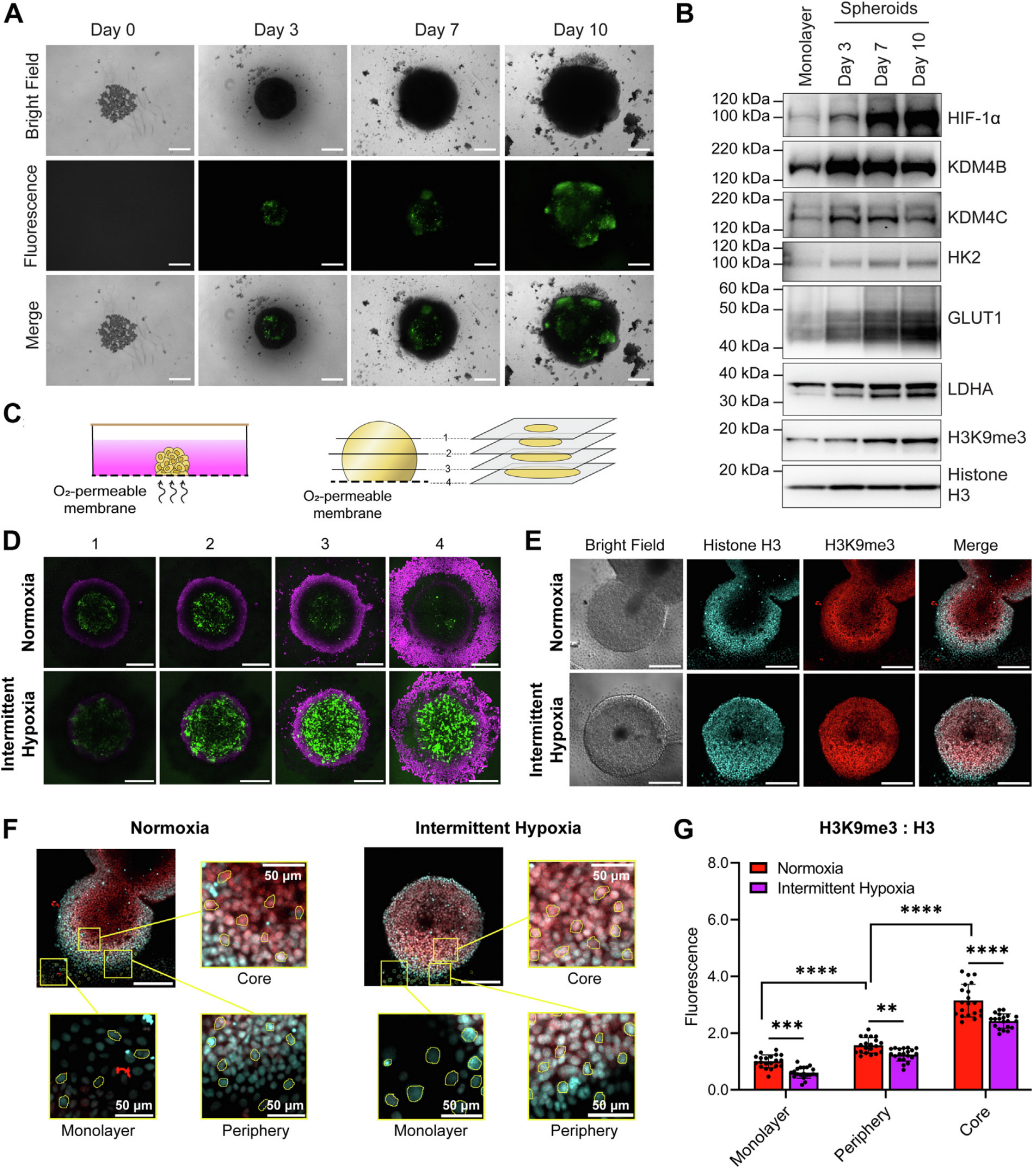
**HIF-1 activity and H3K9me3 levels in a 3D spheroid model of chronic and intermittent hypoxia.***A*, HCT116 cells were stably transfected with a GFP reporter linked to the hypoxia response element (HRE). Cells grown as spheroids and imaged on day 0, 3, 7, and 10. *B*, protein expression of HIF-1α, KDM4B, KDM4C, HK2, Glut1, LDHA, and H3K9me3 in HCT116 cells grown as a monolayer or as spheroids grown over 3, 7, or 10 days. Total histone H3 is used as a loading control. *C*, schematic illustration of spheroid exposure to oxygen conditions using oxygen-permeable membranes and schematic illustration demonstrating how confocal microscopy is used to visualize the spheroid through multiple transverse planes. *D* and *E*, confocal images of HCT116 cells grown as spheroids over 3 days, transferred onto oxygen-permeable membranes for 24 h, and then exposed to normoxia or intermittent hypoxia over a further 18 h. *D*, live cell fluorescence images of spheroids expressing GFP linked to the HRE in four different transverse planes (plane 4 = membrane level). *Magenta* = NucRed live stain; *green* = GFP. *E*, bright field and fluorescence images of spheroids that were fixed, permeabilized, and probed with antibodies. *Cyan* = Histone H3 protein expression; *red* = H3K9me3 protein expression. Images from other transverse planes are shown in Fig. S7. *F*, examples of single cell analysis from individual cells attached to the membrane as a monolayer and from the periphery and core of spheroids (Zoomed in images taken from (*E*)). The scale bar = represents 300 μm for (*A*, *D*, and *E*), 50 μm for (*F*). *G*, fluorescence of H3K9me3:Histone H3 analysis from (*E* and *F*). Results are the mean ± SD of n = 20 cells. ∗∗*p* < 0.01, ∗∗∗*p* < 0.001, ∗∗∗∗*p* < 0.0001. Complete statistical analysis comparing fluorescence between the monolayer *versus* periphery *versus* core is presented in Table S2.

HCT116 cells were transfected with a GFP reporter gene linked to the hypoxia response element (HRE) to which HIF-1 binds. As expected, as spheroids grew larger over time, they developed hypoxic cores with increased HIF-1 activity as indicated by GFP (Fig. 4*A*). GFP expression in the chronically hypoxic spheroid cores was blocked with *HIF1A* siRNA, indicating GFP expression was specific to HIF-1 activity (Fig. S6). The chronic hypoxia generated in the spheroid core increased expression of HIF-target genes, including HK2, LDHA, and GLUT1, as well as increased levels of HIF-1α, KDM4B, and KDM4C proteins, further validating that this model causes regions of chronic hypoxia (Fig. 4*B*). Furthermore, we saw increased H3K9me3 as the spheroid grew in size (Fig. 4*B*). The level of KDM4B, KDM4C, and glycolytic protein (LDHA, GLUT1, and HK2) overexpression and the degree of H3K9me3 correlated with the increase in hypoxia observed *via* GFP (Fig. 4*A*).

Next, we wanted to observe how intermittent hypoxia stemming from the exterior might interact with the chronically hypoxic spheroid core. The 3-day-old HCT116 spheroids stably expressing 5HRE-GFP were placed onto an oxygen-permeable membrane and left to incubate over 24 h to allow cells closest to the membrane to attach (Fig. 4*C*). The face of the spheroid at the membrane has direct exposure to surrounding oxygen environment, including any fluctuations, which mimics the perfused cells on the tumor exterior. As oxygen must diffuse through the spheroid, this leads to spatiotemporal gradients of fluctuating oxygen until it reaches the chronically hypoxic regions at the spheroid core. We wanted to explore how HIF-1 activity would be affected by these spatiotemporal oxygen changes.

Attached spheroids were exposed to either normoxia or intermittent hypoxia over 18 h to assess HIF-1 activity within a three-dimensional cell culture, which introduces additional oxygen gradients. Immediately after exposure, spheroids were stained with nuclear red live (DNA stain) and confocal images were taken through multiple transverse sections of spheroids. This enabled us to examine GFP expression in cells directly attached to the membrane compared to cells deeper into the spheroid, which are more hypoxic due to chronically limited oxygen diffusion. As expected, in normoxia grown spheroids, there was little to no GFP expression in the cells directly attached to the membrane (Fig. 4*D*, Panel 4, top), as the cells are oxygenated. We saw increased GFP expression in the hypoxic core of the normoxic spheroids (Fig. 4*D*, Panel 2 and 3, top), as compared to the oxygenated exterior of the spheroid. When spheroids were placed in intermittent hypoxia, GFP expression was much higher in the cells directly attached to the membrane, confirming that HIF-1 activity increases in cells directly exposed to intermittent hypoxia (Fig. 4*D*, Panel 4, bottom). GFP expression also increased in the core of the intermittent hypoxia spheroids (Fig. 4*D*, Panel 2 and 3, bottom). Interestingly, GFP expression (and therefore HIF-1 activity) was higher in the core of spheroids exposed to intermittent hypoxia than in the spheroids grown in normoxic conditions. This indicates that regions exposed to both chronic and intermittent forms of hypoxia may lead to the greatest HIF-1 activity. These data are supportive of our Western blots in Figure 1*G* showing the highest levels of HIF-1α are found in cells exposed to intermittent hypoxia, followed by chronic hypoxia. Cells may respond to a range of spatially and temporally distinct forms of hypoxia through dynamic or overlapping signaling pathways that combine to enhance activity of HIF-1.

### Intermittent hypoxia decreases H3K9me3 in spheroids

To determine how intermittent hypoxia affects H3K9me3 in an HCT116 spheroid, we used similar conditions as in Figure 4*D*, before fixing spheroids in paraformaldehyde, followed by permeabilization and probing with antibodies for H3K9me3 and total histone (H3). Because antibody penetration can be variable in spheroids even with permeabilization, we used total H3 to account for any bias in antibody penetration. Following confocal microscopy, fluorescence data for both H3K9me3 and total H3 were measured in 20 individual cells from each condition and the ratio of H3K9me3:H3 calculated.

As shown in Figure 4, *E* and *F*, there was a region of single cells grown in monolayer on the membrane surrounding the spheroid. These cells would be exposed to oxygen conditions like those from the monolayers used to produce Western blots in Figure 4*B*. We found that the ratio of H3K9me3:H3 decreased in monolayer cells exposed to intermittent hypoxia, supporting our findings in Figures 1 and 3. When we measured H3K9me3:H3 ratios in cells at the periphery of the spheroids, we also found less H3K9me3 in intermittent hypoxia compared to the periphery of spheroids grown in normoxia (Figs. 4*G* and S7). When we examined H3K9me3:H3 ratios in the core of the spheroids, we saw that H3K9me3 significantly increased as compared to their respective peripheries, which is as expected as chronic hypoxia in the core of the spheroids is likely inhibiting KDM4 activity (Figs. 4*G* and S7).

### Intermittent hypoxia mimicking OSA decreases H3K9me3 and increases *HIF1a* expression in the liver of rats

**Figure 5:**
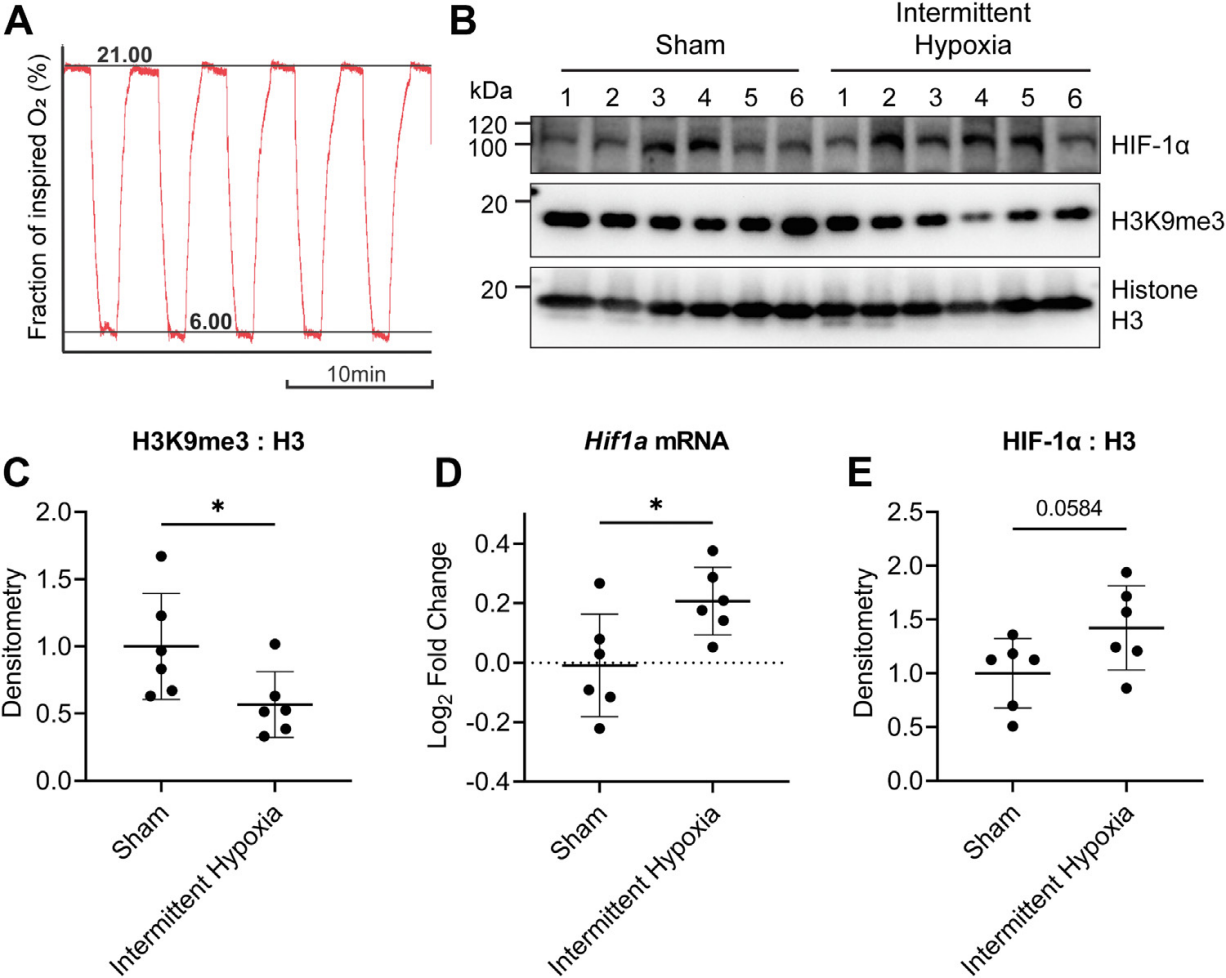
**A rat model of obstructive sleep apnea using intermittent hypoxia leads to H3K9me3 demethylation and increased *HIF1a* expression in the liver.***A*, intermittent hypoxia profile for fraction of inspired oxygen (FiO_2_). During each hypoxic period, the FiO_2_ was reduced from room air levels to 6% O_2_ v/v for 1 min, followed by a reoxygenation to room air (21% O_2_ v/v) for 2.5 min. This protocol was repeated for 8 h. *B*, Western blot of HIF-1α and H3K9me3 levels. Histone H3 is used as a loading control. *C*, densitometry of H3K9me3:Histone H3. *D*, *Hif1a* mRNA levels. *E*, densitometry of HIF-1α:Histone H3. Values are normalized to normoxia (Log2 scale). Results are the mean ± SD of n = 6 rats. ∗*p* < 0.05.

We wanted to test whether intermittent hypoxia would decrease H3K9me3 and increase *HIF1A in vivo*. We had access to frozen liver tissue from a Sprague–Dawley rat model of OSA. Rats were previously exposed to 8 h of cycling intermittent hypoxia *via* inhaled gases (oxygen trace shown in Fig. 5*A*), which generates fluctuating blood oxyhemoglobin levels and intermittent hypoxia within the liver and other tissues (21). This fluctuating pattern of intermittent hypoxia is designed to mimic the systemic intermittent hypoxia observed in patients with OSA (22). We obtained the liver tissues and measured H3K9me3 and compared to control rats exposed to circulating inhaled air with no change in oxygen levels. We found that H3K9me3 decreased in rats exposed to intermittent hypoxia (Fig. 5, *B* and *C*), supporting our results in Figure 1. Furthermore, *HIF1a* mRNA increased in the livers of rats exposed to intermittent hypoxia (Fig. 5*D*), indicating that systemic intermittent hypoxia can lead to H3K9me3 demethylation and an increase in expression of *HIF1a* in the liver. We also measured HIF-1α levels and saw a trend toward increased HIF-1α in rats exposed to intermittent hypoxia (Fig. 5, *A* and *E*, *p* value = 0.058).

## Discussion

**Figure 6:**
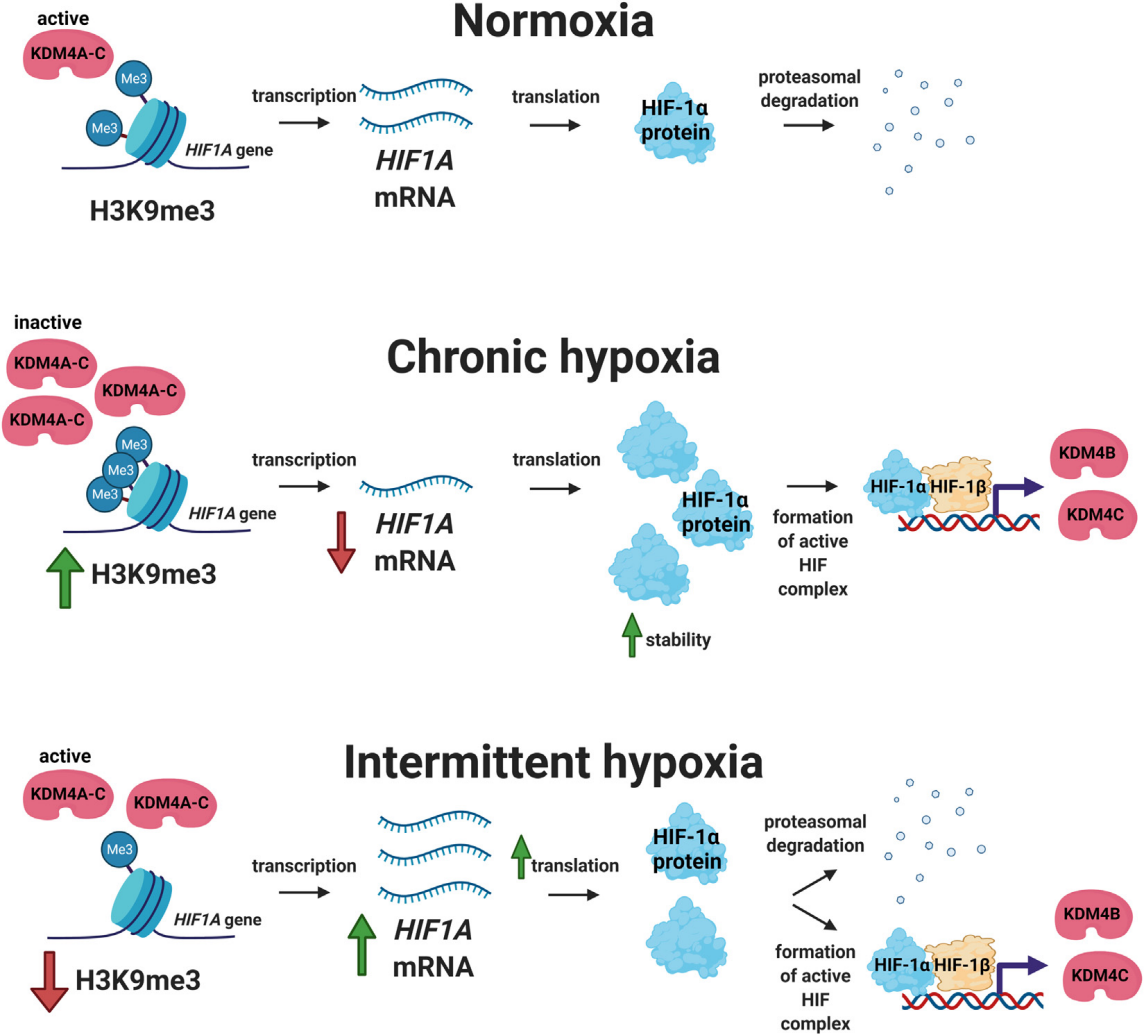
**Proposed mechanism of HIF-1 activation in intermittent *versus* chronic hypoxia.** In normoxia, *HIF1A* is constitutively transcribed and translated into HIF-1α but HIF-1α is posttranslationally degraded. In chronic hypoxia, HIF-1α is stabilized, increasing HIF-1 transcriptional activity and the expression of HIF target genes, KDM4B and KDM4C. Despite increased enzyme levels, KDM4A, KDM4B, and KDM4C are largely inactive due to limited amounts of oxygen, which are required for KDM activity. This leads to an increase in H3K9me3, including at the *HIF1A* locus, which ultimately decreases the amount of *HIF1A* mRNA being transcribed. In intermittent hypoxia, HIF-1α increases as compared to normoxia. KDM4B and KDM4C expression levels increase somewhat but not to the same level as chronic hypoxia. However, in contrast to chronic hypoxia, KDM4A, KDM4B, and KDM4C activity increases due to sufficient oxygen and higher levels of demethylases, leading to higher levels of H3K9 demethylation at the *HIF1A* gene when compared to normoxia or chronic hypoxia. This results in increased *HIF1A* mRNA production.

Our study reveals a novel oxygen-sensing mechanism that is differentially regulated depending on whether cells are exposed to intermittent *versus* chronic hypoxia (minutes *versus* hours) (Fig. 6). In agreement with previous studies, we found that chronic hypoxia increases HIF-1α and globally increases H3K9me3. These effects are known to be due to inactivation of oxygen sensors (23), namely the PHDs and KDM4s, respectively. Traditionally, intermittent hypoxia, including the rapid form which is associated with OSA, is regarded as having the same effect on oxygen-sensing pathways as chronic hypoxia. However, our results using minutes of intermittent hypoxia reveals different effects on total KDM4 activity when compared to hours of chronic hypoxia. We found that intermittent hypoxia decreases H3K9me3 by increasing the quantity and demethylation turnover of KDM4 enzymes. KDM4 specifically demethylates the *HIF1A* gene locus, so increased KDM4 activity and quantity ultimately has consequences for HIF-1 activity through increased *HIF1A* gene expression, demonstrating a unique relationship between the two oxygen-sensing pathways in intermittent hypoxia. New oxygen-sensing pathways have been identified very recently, including multiple KDMs (9, 10, 11) and ADO (24), and there are a limited number of studies looking at how multiple oxygen-sensing pathways might interact under different forms of hypoxia. It is likely that mechanisms and systems have evolved between the oxygen-sensing pathways that enable the cell to fine tune the response to hypoxia, whether it is in the form of acute, chronic, or intermittent, and based on the severity of hypoxia.

Our results are intriguing when viewed in light of studies that focus on the ‘preconditioning effects’ of sleep apnea and cardiovascular disease. Intermittent hypoxia from mild to moderate sleep apnea has been suggested to have a protective effect against myocardial infarction in both humans and animal models (25, 26, 27, 28, 29, 30). Sleep apnea patients have higher levels of serum erythropoietin (EPO) during acute myocardial infarction (31), and EPO expression is largely controlled by HIF (32), indicating that sleep apnea may increase HIF activation. Increased stabilization of HIF is beneficial in myocardial outcomes (33). However, studies indicate that while enhanced cardiac expression of HIF-1α is beneficial, protection requires activation before the onset of lethal ischemia (34). Our cellular model of intermittent hypoxia induces a significant increase in *HIF1A* mRNA expression, though the full effects of this increase are not immediately reflected at the protein level due to competing HIF-1α degradation controlled by the PHDs. However, when degradation of HIF-1α is halted, either by a drug (MG262, Fig. 1*F*) or through an additional period of chronic hypoxia (Fig. 1*G*), the surplus of *HIF1A* mRNAs is converted into protein, with little degradation, resulting in a corresponding large increase in HIF activity. In this unique situation, an abundance of *HIF1A* mRNA could prove to be beneficial during ischemia by enabling maximum activation of the HIF-1 pathway. While our studies did not use cardiac cells or ischemia models, this is an intriguing hypothesis to be tested in future.

Additionally, our spheroid model showed that the HIF-1 response to chronic hypoxia can be further impacted by intermittent hypoxia. Chronic hypoxia in the spheroid center led to HIF-1 activation, and when combined with intermittent hypoxia on the exterior of the spheroid, HIF-1 activation was greatly enhanced both in the center of the spheroid and closer to the periphery. H3K9me3 levels were overall lower in spheroids exposed to intermittent hypoxia and it is likely that different oxygen-sensing pathways may behave differently depending on the type(s) of hypoxia (intermittent *versus* chronic) in the different regions (periphery *versus* core) of the spheroid. Several studies have suggested that fluctuating oxygen levels in tumors lead to greater phenotypic variation and a competitive advantage for tumors (35, 36). This could be one way that intermittent hypoxia alters tumor behavior. Furthermore, enhanced KDM4 activity or increased levels of KDM4B and/or KDM4C caused by intermittent hypoxia may also impact tumors, as the KDM4 group is associated with various cancers (37).

One of the limitations to our study is the use of a small number of patterns of intermittent hypoxia. Studying the biological response to intermittent hypoxia is difficult because the pattern of intermittent hypoxia *in vivo* varies widely. We chose to primarily focus on periods of hypoxia lasting 5 to 8 min and occurring multiple times per hour for two reasons. One, these periods of hypoxia are more closely relevant to fluctuating tumor hypoxia and sleep apnea, and two, these periods of hypoxia are achievable in cell cultures using oxygen permeable membranes. As shown in Fig. S8, altering the lengths of normoxia and hypoxia in cell cultures lead to different peak and nadir oxygen values as measured at the pericellular level. This is despite the oxygen concentration in the air surrounding the dishes remaining the same. This is likely due to the slow diffusion of oxygen in and out of the cell culture media and presents limitations in experimental setup. Furthermore, in designing an intermittent hypoxia protocol to mimic physiological hypoxia, it is difficult to determine the ‘severity’ of intermittent hypoxia *in vivo*. It is not clear how depth of hypoxia, number of cycles, rapidity of cycles, or dose of hypoxia will affect the pathways identified in intermittent hypoxia, and further study into how the dynamics of oxygen deprivation and reoxygenation impact cellular pathways is needed. Whether the response we identified in this study occurs in all forms and lengths of intermittent hypoxia is yet to be tested. Future studies will help us to gain further understanding into how new cellular oxygen sensors respond and interact in intermittent hypoxia to generate the hypoxic response.

## Experimental procedures

### Cell culture

All cell lines were maintained in media supplemented with 10% fetal bovine serum (FBS), 1× Glutamax, penicillin (50 IU/ml), and streptomycin (50 μg/ml). HCT116 cells were cultured in McCoys 5A media (Gibco). MCF7, MDA-MB-231, and U251 cells were cultured in Dulbecco's modified Eagle's medium (DMEM) media (Gibco). PC3 cells were cultured in DMEM/F12 media (Gibco). Cell cultures were *mycoplasma* tested a minimum of four times per year.

### Cell culture model of intermittent hypoxia

To overcome the challenges of slow oxygen diffusion through culture media, cells were grown onto oxygen-permeable polydimethylsiloxane (PDMS) membranes to enable cells to access oxygen from the ambient air directly through the membrane. PDMS culture dishes were purchased from Sarstedt, Zell-Kontakt, or Coy Lab Products. Cells were seeded onto PDMS culture dishes in media supplemented with Glutamax and 10% FBS. After overnight incubation, the media was replaced with fresh media with 0.5% FBS. Cells were placed in hypoxia chambers and exposed to different oxygen conditions, as defined later. Construction of hypoxia chambers are described in (38). Oxygen levels in the air surrounding cell culture dishes was controlled using gas blenders (3 channel GB100 from MCQ Instruments) connected to piped carbon dioxide, oxygen, and nitrogen gases. The 5% carbon dioxide was used in all gas mixtures for buffering purposes, while oxygen fraction was varied depending on desired hypoxia conditions.

#### Defining normoxia and chronic hypoxia

Exposure to oxygen conditions was carried out as previously described in detail (12, 38). Normoxia was set to 89 mmHg (12% v/v) to reflect physiological tissue oxygenation near major blood vessels (3). Unless specified otherwise, chronic hypoxia was set to 4 mmHg (0.5% v/v) for 18 h to reflect hypoxia in a tumor (3). Some experiments used 15 mmHg (2% v/v), which is also relevant to tumor biology (3), and mimics previously used conditions to provide direct comparison when studying H3K9 methylation at the *HIF1A* gene (15). All experiments using 2% v/v conditions are indicated in the relevant figures.

#### Defining intermittent hypoxia

Many studies of intermittent hypoxia use room air for peak oxygenation levels, which does not reflect *in vivo* oxygen conditions. To more accurately represent physiology, we based our oxygen concentrations for intermittent hypoxia on measurements taken in a tumor in an animal model of OSA (39). Oxygen fluctuations in the tumor alternated between ∼5 to 50 mmHg, so our oxygen cycling protocols were modified to produce fluctuations between 5 and 50 mmHg in the pericellular media. We exposed cells to a normoxic phase of 8% O_2_ v/v and a hypoxic phase of 0% O_2_ v/v and altered the lengths of time in the normoxic and hypoxic phase. Cells were exposed to 3 min normoxia followed by 3 min of hypoxia (3 min/3 min), 5 min/5 min, 8 min/8 min, or 12 min/12 min. Altering the periods of normoxia and hypoxia led to different peak and nadir oxygen values in the pericellular media as measured using a PreSens Oxygen Microsensor oxygen electrode (PM-PSt-7 with Oxy-1 ST) (Fig. S8). For the primary protocol of intermittent hypoxia, we exposed cells to 5 min/5 min or 8 min/8 min, as these protocols induced the greatest changes in histone methylation. Cells were exposed to these oxygen conditions over 18 h unless specified otherwise.

### Treatment with CHX

Cells were seeded onto PDMS dishes. After overnight incubation, media was removed and replaced with fresh media with 0.5% FBS containing 10 μg/ml CHX (C1988, Sigma–Aldrich) or vehicle control (1% dimethyl sulfoxide). Dishes were then exposed to normoxia, chronic hypoxia, or intermittent hypoxia for 6 h.

### Treatment with proteasome inhibitor

Cells were seeded onto PDMS dishes. After overnight incubation, media was removed and replaced with fresh media with 0.5% FBS containing 1 mM MG262 (Calbiochem) or vehicle control (1% dimethyl sulfoxide). Dishes were then exposed to normoxia, chronic hypoxia, or intermittent hypoxia for 18 h.

### siRNA

Cells were transfected with siRNA using HiPerfect (Qiagen). We used 20 nM KDM4A FlexiTube GeneSolution siRNA (Qiagen, GS9682), 5 nM siKDM4B (Qiagen, SI00449764), 5 nM siKDM4C (Qiagen, SI05163977), and 20 nM HIF1A Flexitube GeneSolution siRNA (Qiagen, GS3091). AllStars Negative control siRNA (Qiagen, SI03650318) was used as a negative control. Cells incubated for 48 h prior to exposure to normoxia, chronic hypoxia, and intermittent hypoxia. Immediately prior to exposure to oxygen conditions, media was replaced with fresh media supplemented with 0.5% FBS.

### Western blotting

To extract total protein, cells or tissues were lysed in radioimmunoprecipitation assay buffer. To extract nuclear protein, nuclear lysates from cells or tissues were prepared as described previously (38). Tissues were homogenized using a 5 mm stainless steel bead (Qiagen) and TissueLyser LT (Qiagen). Cell or tissue lysates were briefly sonicated to shear DNA. Lysates were mixed in 4× LDS loading buffer containing DTT (ThermoFisher) and heated to 70 °C for 10 min. Proteins were separated by SDS-PAGE and resolved using semidry transfer. Polyvinylidene difluoride membranes were blocked in 5% skim milk. Antibodies used were as follows: HIF-1α (NB100-479, 1:500, Novus), Tri-Methyl-Histone H3 Lys9 (13969, 1:1000, Cell Signaling Technologies), Mono-Methyl-Histone H3 Lys9 (14186S, 1:1000, Cell Signaling Technologies), Di-Methyl-Histone H3 Lys9 (4658S, 1:1000, Cell Signaling Technologies), Total Histone H3 (ab1791, 1:2000, Abcam), Hexokinase II (TA325030, 1:500, Origene), Glut1 (ab115730, 1:1000, Abcam), LDHA (3582T, 1:1000, Cell Signaling Technologies), JMJD2A (5328, 1:1000, Cell Signaling Technologies), JMJD2B (8639, 1:1000, Cell Signaling Technologies), JMJD2C (ab226480, 1:500, Abcam).

### Quantitative RT-PCR

Total RNA was extracted using the RNeasy Mini Kit (Qiagen). RNA was quantified using a Nanodrop (Molecular Devices). Total RNA (2 μg) was used as a template for complementary DNA (cDNA) synthesis using the High-Capacity cDNA Reverse Transcription Kit (Applied Biosystems). For quantitative RT-PCR, predesigned TaqMan Gene Expression assays were used with TaqMan Gene Expression Master Mix (Life Technologies, ThermoFisher). Twenty nanograms cDNA was used per reaction. qPCR was performed using a QuantStudio 7 Flex Real-Time PCR System. Relative gene expression was normalized to eukaryotic 18S rRNA (Hs03003631_g1) or ACTB (Hs01060665_g1) and fold change was calculated using the ΔΔCt method. Target gene TaqMan primers (Assay ID) used for qPCR (ThermoFisher) were as follows: SLC2A1 (Hs00892681_m1), HK2 (Hs00606086_m1), LDHA (Hs01378790_g1), PLOD2 (Hs01118190_m1), P4HA1 (Hs00914594_m1), P4HA2 (Hs00990001_m1), EGLN1 (Hs00254392_m1), EGLN3 (Hs00222966_m1), HIF1A (Hs00153153_m1), KDM4A (Hs00206360_m1), KDM4B (Hs00392119_m1), and KDM4C (Hs00909577_m1). Data are expressed as the mean ± SEM from three or more independent experiments.

### KDM4 enzyme activity assay

KDM4 demethylase activity was measured using the JMJD2/KDM4 Activity Quantification Assay Kit (ab113461, Abcam) according to the manufacturer’s instructions. Recombinant KDM4A (31857, Active Motif), KDM4B (ab198159, Abcam), or KDM4C (ab198094) were added to wells coated with H3K9me3 in a range of concentrations (0–800 ng). Reactions took place in the same hypoxic chambers used for cell cultures under normoxia, chronic hypoxia, and intermittent hypoxia over 4 h. Demethylated H3K9me3 were captured using a primary antibody overnight. Wells incubated in a horseradish peroxidase–linked secondary detection antibody for 2 h. Absorbance readings were taken at 450 nm with a reference wavelength of 655 nm using the Infinite M1000 PRO microplate reader (TECAN).

### Spheroids

HCT116 cells were transfected with 5 μg 5HRE/GFP plasmid, a gift from Martin Brown & Thomas Foster (Addgene plasmid #46926; http://n2t.net/addgene:46926; RRID:Addgene_46926) (40) using lipofectamine 3000 (ThermoFisher). Stably expressing cells were selected for using G418 (400 μg/ml) to create a stable HCT116-5HRE/GFP cell line. To form multicellular tumor spheroids, HCT116-5HRE/GFP cells were seeded onto a 96-well clear round bottom ultralow attachment plate (Costar) and spheroid growth was monitored over 3, 7, and 10 days. For Western blots, approximately 60 spheroids were collected on day 3, day 7, and day 10 and lysed in radioimmunoprecipitation assay buffer. For confocal images, stably transfected HCT116-5HRE/GFP spheroids grown over 3 days were placed onto oxygen-permeable membranes and incubated for a further 24 h to allow spheroids to attach the membrane prior to exposure to normoxia or intermittent hypoxia for 18 h. Spheroids were then stained with NucRed Live 647 ReadyProbes Reagent (R3106, ThermoFisher) 30 min prior to live cell imaging. For H3K9me3 expression, regular HCT116 cells were plated as spheroids, grown over 3 days, and exposed to normoxia and intermittent hypoxia as aforementioned. Spheroids were then fixed and permeabilized for 2 h in PBS containing 4% paraformaldehyde, and 1% Triton X-100. Spheroids were dehydrated in an ascending series of methanol (25%, 50%, 75%, 95% 20 min each, and 100% for 2 h) and then rehydrated in the same descending series of methanol (41). Spheroids were blocked in 3% bovine serum albumin overnight, incubated with primary antibodies over 48 h, and with secondary antibodies over 24 h. Antibodies used were as follows: tri-methyl-histone H3 Lys9 (13969, 1:50, Cell Signaling Technologies) and total histone H3 (14269S, 1:50, Cell Signaling Technologies), anti-rabbit IgG Alexa Fluor 488 conjugate (4408S, 1:50, Cell Signaling Technologies), antimouse IgG Alexa Fluor 647 conjugate (4414S, 1:50, Cell Signaling Technologies).

### Microscopy (confocal and regular fluorescence)

Fluorescence images were acquired using a Zeiss Primovert microscope with 5× and 10× objective. Live cell confocal images for GFP expression were acquired using a Leica TCS SP8 confocal laser scanning microscope with 25 × W 0.95 objective. Three-dimensional images were collected and stitched using LASX Navigator module. Confocal imaging of fixed spheroids labeled with H3K9me3 and Histone H3 was performed using a Zeiss LSM 880 microscope with 20× air objective, NA 0.8. For analysis, 60 μm z-stacks (step size 15 μm) were acquired using Zen software (black version 2.3, Carl Zeiss Microscopy GmbH). Images were processed and analyzed using Fiji ImageJ (42). Single cells from the core and periphery of the spheroids and from individual cells attached to the membrane were selected (see Fig. 4*F*) and fluorescence of H3K9me3 and Histone H3 was quantified.

### Chromatin immunoprecipitation

Protein was crosslinked to chromatin with 1% formaldehyde for 10 min. Nuclear extracts were prepared by douncing lysates in swelling buffer (25 mM Hepes, pH 8, 1.5 mM MgCl_2_, 10 mM KCl, 0.1% NP-40, 1 mM DTT, EDTA-free protease inhibitors). Nuclei were resuspended in sonication buffer (50 mM Hepes, pH 8, 140 mM NaCl, 1 mM EDTA, pH 8, 1% Triton X-100, 0.5% Na-deoxycholate, 0.1% SDS, EDTA-free protease inhibitors) and sonicated at 70% amplitude for 40 min, (15 s ON, 45 s OFF) at 4 °C (QSonica 800R) to shear DNA to an average fragment size of 200 to 1000 bp. Prior to immunoprecipitation, 10% of each lysate was aliquoted to be used as an input reference sample. The remaining lysates were diluted 1:10 in dilution buffer (50 mM Tris–HCl, pH 8, 150 mM NaCl, 2 mM EDTA, pH 8, 1% NP-40, 0.5% Na-deoxycholate, 0.1% SDS, EDTA-free protease inhibitors) and incubated in 10 μg H3K9me3 antibody (1369, Cell Signaling) overnight at 4 °C. Immune complexes were captured by incubation with Protein G Dynabeads (Thermo) at 4 °C for 2 h. Dynabeads were washed sequentially with a low salt wash buffer (20 mM Tris–HCl, pH 8, 150 mM NaCl, 2 mM EDTA, 1% Triton X-100, 0.1% SDS), a high salt wash buffer (20 mM Tris–HCl, pH 8, 500 mM NaCl, 2 mM EDTA, 1% Triton X-100, 0.1% SDS), a LiCl wash buffer (10 mM Tris–HCl, pH 8, 250 mM LiCl, 1 mM EDTA, 1% NP-40, 1% Na-deoxycholate), followed by TE buffer (10 mM Tris–HCl, pH 8, 1 mM EDTA), and eluted with 120 μl elution buffer (1% SDS, 100 mM NaHCO_3_). Crosslinks were reversed by incubation with 0.2 M NaCl and 10 mg/ml RNase A at 65 °C for 4 h. Proteins were digested by incubation with 20 mg/ml proteinase K for 1 h at 60 °C. DNA was purified using the Monarch PCR & DNA Cleanup Kit (NEB), according to the manufacturer’s instructions. Immunoprecipitated DNA was quantified by qPCR relative to the total amount of input DNA. Primer sequences for introns on the *HIF1A* gene are described in (15).

### Rat intermittent hypoxia model

#### Animals

Experiments were conducted with approval from the Animal Care and Ethics Committee of the Sydney Local Health District. Procedures were conducted in accordance with the Australian Codes of Practice for the Care and Use of Animals for Scientific Purposes (New South Wales: Animal Research Act 1985, 8th edition 2013). All experiments were conducted on adult male Sprague–Dawley rats weighing 300 to 500 g, housed three per cage in the Heart Research Institute biological facilities animal holding room. Rats acclimatized in the holding room for at least 7 days before experiments and lived in a 12/12 h light-dark schedule with lights on at 0700 AM. Room temperature was set at 22 ± 2 °C with relative humidity of 40% to 60%. Food and water were available ad libitum.

#### Experimental design

Twelve rats were randomly allocated to either an intermittent hypoxia cohort or a control cohort. The intermittent hypoxia or Sham protocol was started between 8 to 9 AM. Food was removed during the 8 h intermittent hypoxia period but water remained ad libitum.

#### Intermittent hypoxia protocol and liver collection

Rats were individually exposed to 8 h of intermittent hypoxia designed to mimic blood oxygen changes seen in OSA or to a Sham sequence inside a modified home cage. High purity nitrogen and oxygen (Coregas) was mixed at the desired percentages by a GSM-4 Gas Mixer (CWE Inc). Oxygen levels (% O_2_) within the chamber were continuously monitored (OxyStar-100 Oxygen Monitor, CWE Inc) and recorded in Spike2 software (Ver.8 software CED Ltd). Intermittent hypoxia was delivered in 4-phase cycles of varying oxygen and nitrogen flow rates. Oxygen levels were rapidly driven from 21% down to 6% then sustained for 1 min. Oxygen was then rapidly ramped back up to 21% and maintained for 2.5 min. This sequence was repeated for 8 h. The timed sequence and flow rates for the Sham cohort exactly matched those of the intermittent hypoxia sequence but with 21% O_2_ delivered across all four phases. Following the 8 h protocol, rats were anaesthetized with sodium pentobarbitone (90 mg/kg, Lethabarb Euthanasia Injection, Virbac Pty, Ltd) and blood collected *via* cardiac puncture. Rats were transcardially perfused with ice-cold PBS (400 ml). The right median lobe of the liver was extracted and immediately freeze clamped in liquid nitrogen. The resultant wafer was stored at −80 °C for future use.

### Statistics

Statistics were conducted using GraphPad Prism (GraphPad Software Inc). One-way ANOVA was used when comparing results between normoxia, chronic hypoxia, and intermittent hypoxia. Paired two-tailed *t* test was used to compare siCONTROL with siHIF1A or siKDM4. Two-way ANOVA was used for KDM4B and KDM4C enzyme assays at varying concentrations (see Table S1) and for H3K9me3:H3 expression in spheroids (see Table S2). Mann–Whitney U test was used for all rat liver data. The number of biological replicates for each experiment is described in the figure legends.

## Data availability

All data are contained in the article.

## Supporting information

This article contains supporting information (38).

## Conflicts of interest

The authors declare that they have no conflicts of interest with the contents of this article.
