# Supplementary material for: Intermittent hypoxia enhances the expression of hypoxia inducible factor HIF1A through histone demethylation
Source: J Biol Chem. 2022 Sep 27;298(11):102536. doi: 10.1016/j.jbc.2022.102536 (PMC9597902; doi:10.1016/j.jbc.2022.102536)
Supplement: Supporting Information [file mmc1.docx]

Intermittent hypoxia enhances the expression of HIF1A through histone demethylation

Chloe-Anne Martinez^1^, Yannasittha Jiramongkol^2^, Neha Bal^1^, Imala Alwis^1,3^, Polina Nedoboy^1,3^, Melissa Farnham^1,3^, Mark White^2^, Peter A Cistulli^1^, and Kristina M Cook^1*^

^1^University of Sydney, Faculty of Medicine and Health, Charles Perkins Centre, Camperdown, NSW 2006 Australia

^2^University of Sydney, Faculty of Science, Charles Perkins Centre, Camperdown, NSW 2006 Australia

^3^Heart Research Institute, Sydney, NSW

^*^Corresponding author: [kristina.cook@sydney.edu.au](mailto:kristina.cook@sydney.edu.au)

**Supporting Information**

**Supplemental Data:**


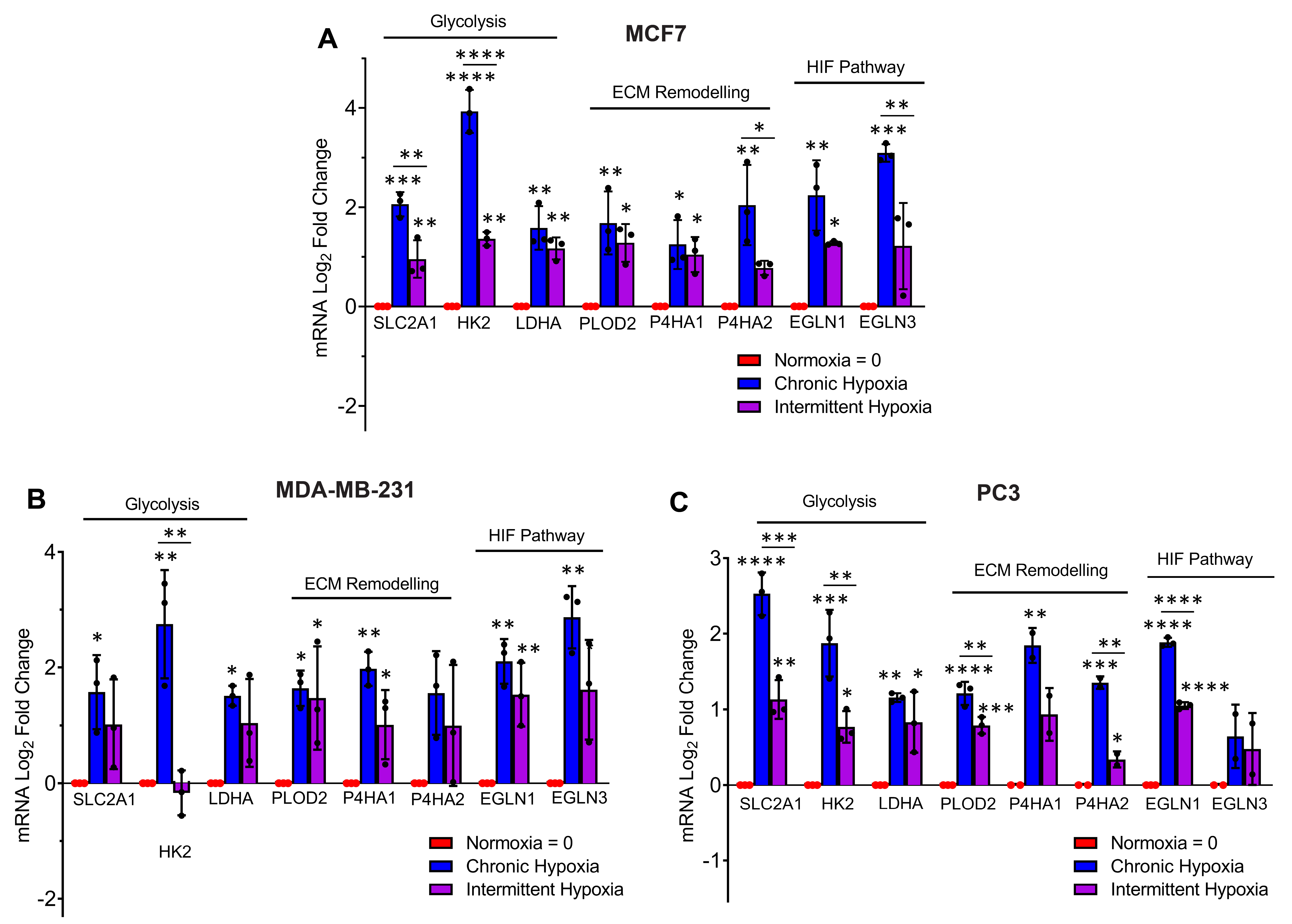


**Supplemental Figure 1.** mRNA expression of HIF-1 target genes involved in glycolysis (*SLC2A1, HK2, LDHA*), extracellular matrix remodeling (*PLOD2, P4HA1, P4HA2*) and the HIF pathway (*EGLN1, EGLN3*) in (A) MCF7, (B) MDA-MB-231, and (C) PC3 cells exposed to normoxia, chronic hypoxia and intermittent hypoxia (5 min/5 min). Expression increased in all tested cell lines exposed to chronic or intermittent hypoxia. HK2 (hexokinase 2) decreased in MDA-MB-231 cells exposed to intermittent hypoxia, while increasing in MCF7 and PC3 cells. The reasons for these discrepancies are not clear but may be due to cell line-specific gene regulation differences or mutations. Values are normalized to normoxia (Log2 scale). Mean ± S.D. of n = 3. * p < 0.05, ** p < 0.01, *** p < 0.001, **** p < 0.0001. Asterisks above a data point (without line) indicate significance as compared to normoxia. Asterisks above a line compare data points connected by the line.


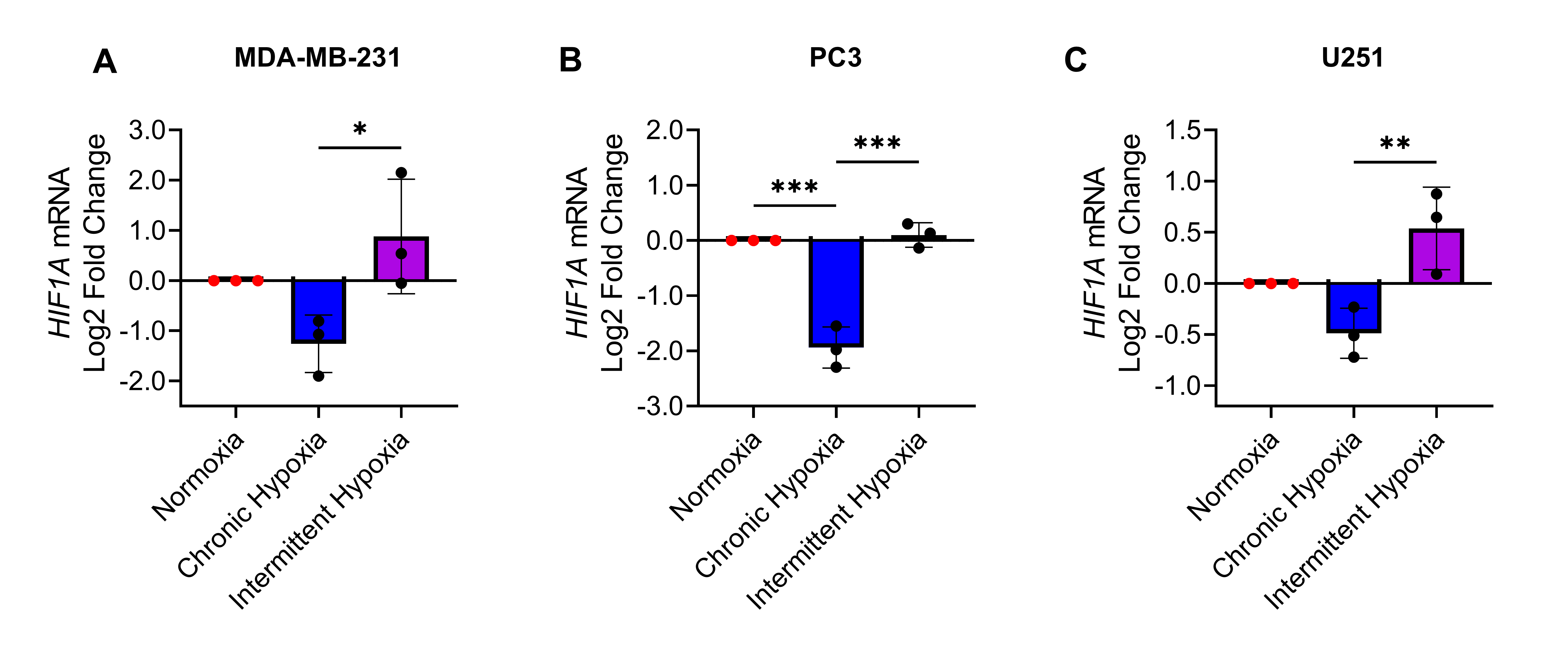


**Supplemental Figure 2.** *HIF1A* mRNA expression decreases in chronic hypoxia and increases in intermittent hypoxia in (A) MDA-MB-231, (B) PC3, and (C) U251 cells. Cells were exposed to normoxia, chronic hypoxia and intermittent hypoxia (5 min/5 min). Values are normalized to normoxia (Log2 scale). Mean ± S.D. of n = 3.


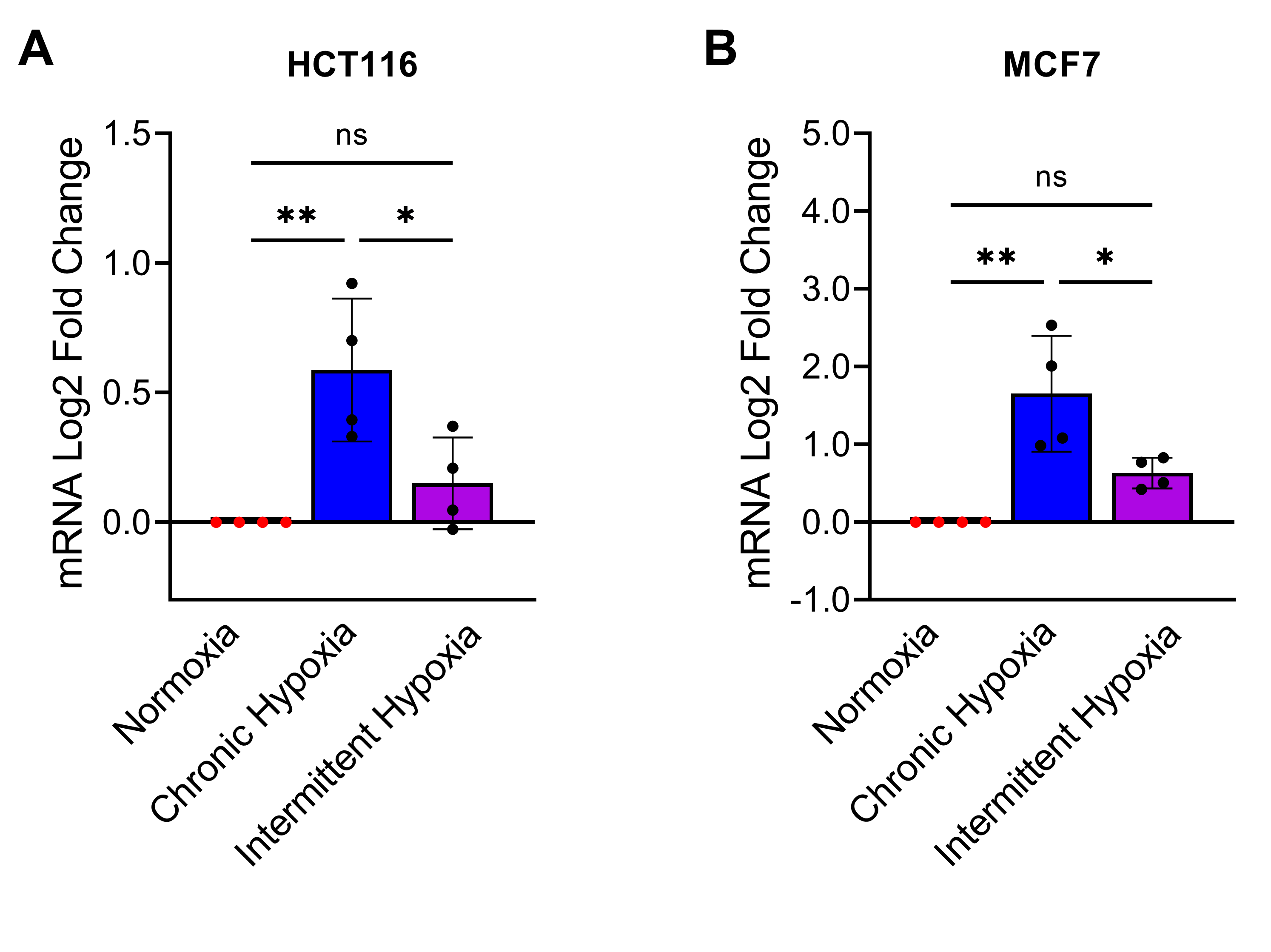


**Supplemental Figure 3.** *EPAS1 (HIF2A)* mRNA expression increases in chronic and intermittent hypoxia in (A) HCT116 and (B) MCF7 cells. Cells were exposed to normoxia, chronic hypoxia and intermittent hypoxia (5 min/5 min). Values are normalized to normoxia (Log2 scale). Mean ± S.D. of n = 4.

**
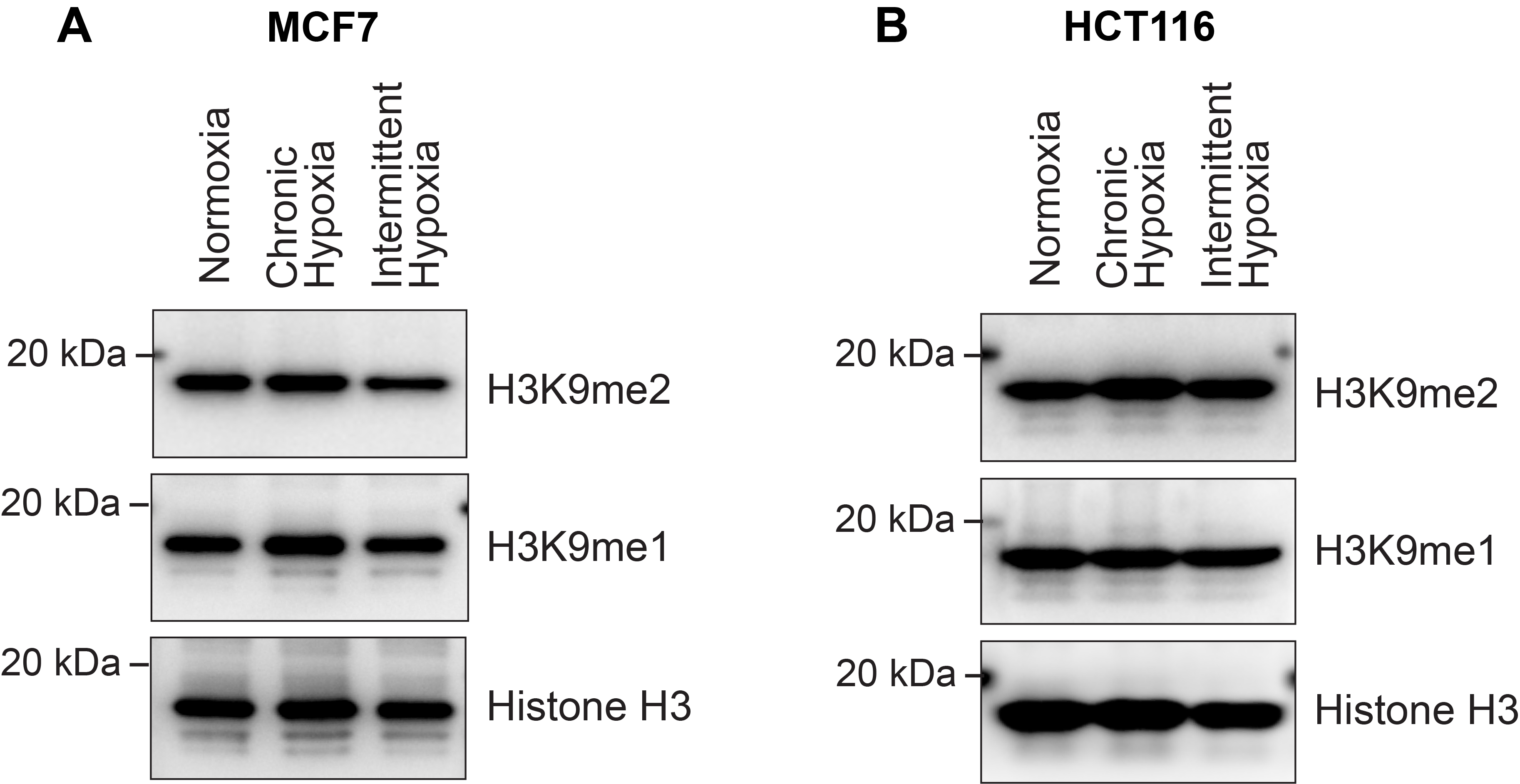
**

**Supplemental Figure 4.** H3K9me2 and H3K9me1 levels in (A) MCF7 cells and (B) HCT116 cells exposed to normoxia, chronic hypoxia and intermittent hypoxia (5 min**/**5 min). Histone H3 is used as a loading control.


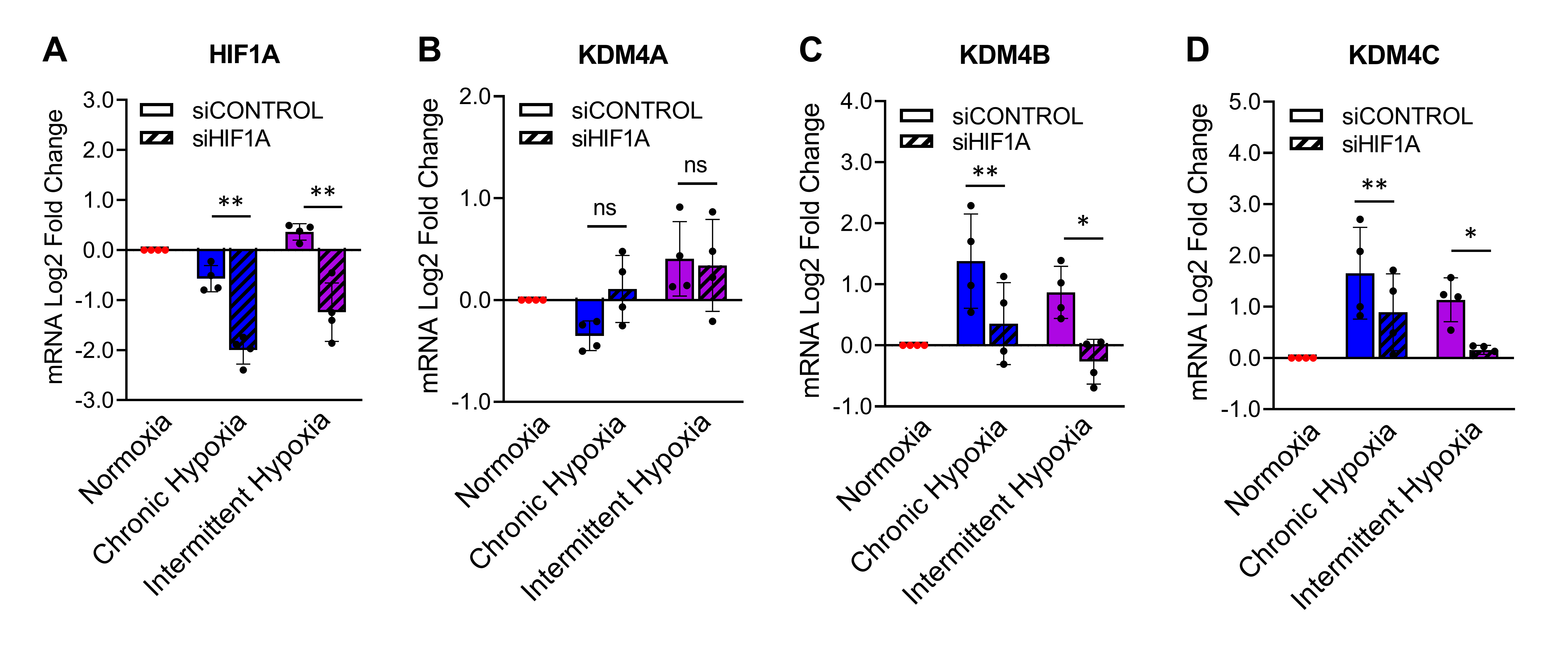


**Supplemental Figure 5.** mRNA levels of (A) *HIF1A*, (B) *KDM4A*, (C) *KDM4B*, and (D) *KDM4C* in MCF7 cells transfected with *HIF1A* siRNA (siHIF1A) or scrambled control siRNA (siCONTROL) prior to exposure to normoxia, chronic hypoxia and intermittent hypoxia (5 min/5 min). Values are normalized to normoxia (Log2 scale). Results are the mean ± S.D. of n = 4. ns = not significant, * p < 0.05, ** p < 0.01, *** p < 0.001, **** p < 0.0001.

**
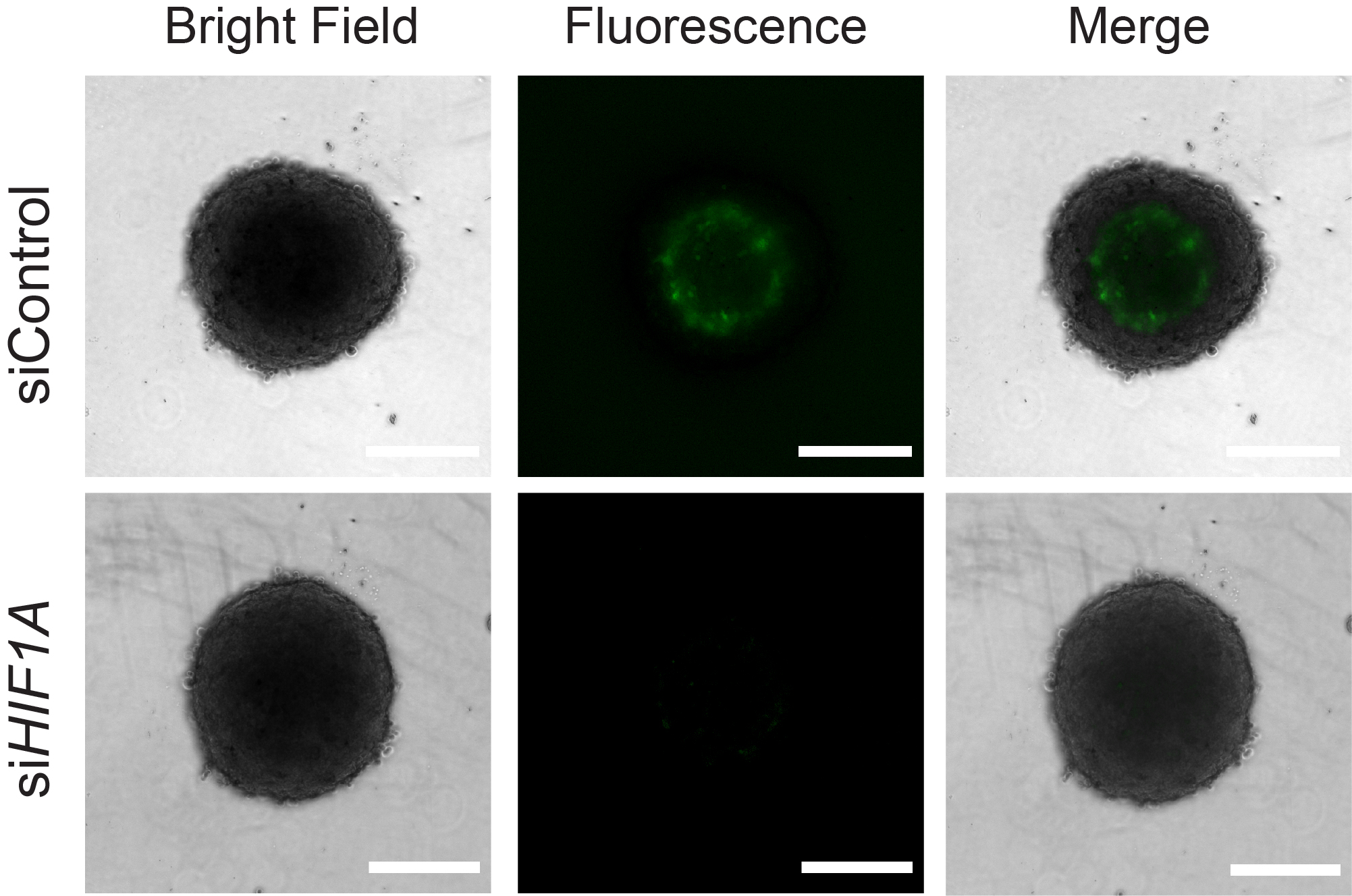
**

**Supplemental Figure 6.** Bright field and fluorescence images of HCT116 spheroids expressing a GFP reporter linked to the hypoxia response element transfected with scrambled control siRNA (siControl) or *HIF1A* siRNA (siHIF1A). Scale bar = 300 µm.


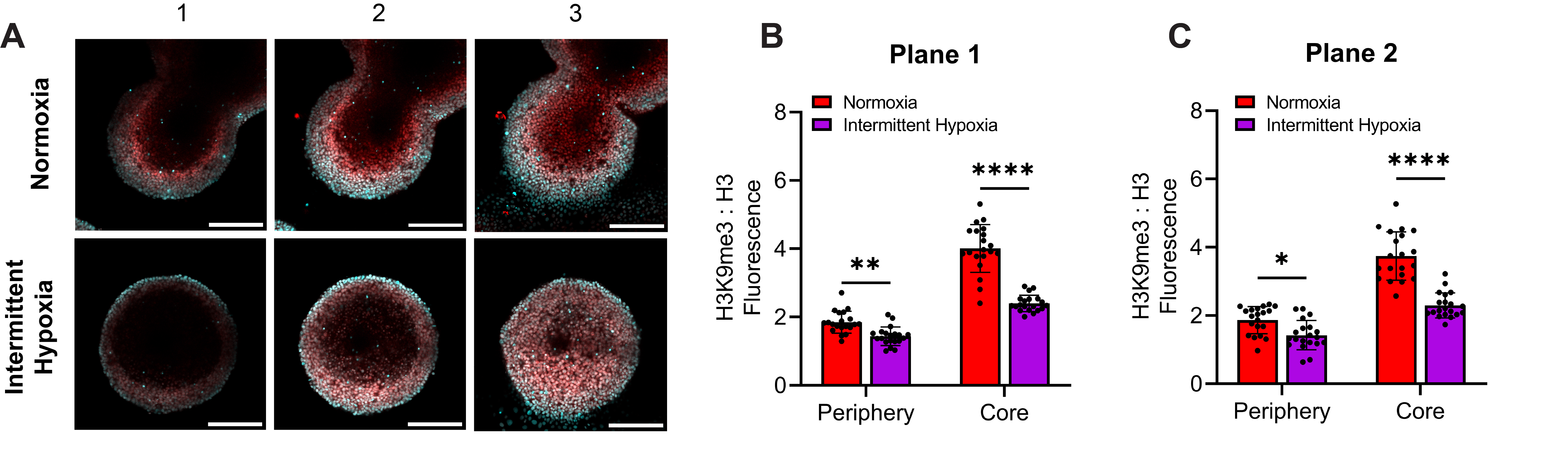


**Supplemental Figure 7.** Confocal images of H3K9me3 and Histone H3 expression in HCT116 spheroids exposed to normoxia and intermittent hypoxia (5 min/5 min). (A) Spheroids were fixed, permeabilized and probed with antibodies and imaged in three different transverse planes (Plane 1 = deepest layer, Plane 2 = middle layer, Plane 3 = membrane level). Cyan = Total Histone H3 protein expression; Red = H3K9me3 protein expression. Scale bar = 300 µm. (B) Fluorescence intensity of H3K9me3:Histone H3 at plane 1. (C) Fluorescence intensity of H3K9me3:Histone H3 at plane 2. Fluorescence intensity of H3K9me3:Histone H3 of plane 3 is shown in Figure 4. Results are the mean ± S.D. of n = 20 cells. * p < 0.05, ** p < 0.01, **** p < 0.0001. Complete statistical analysis comparing fluorescence between the periphery and core is presented in Supplemental Table 2.


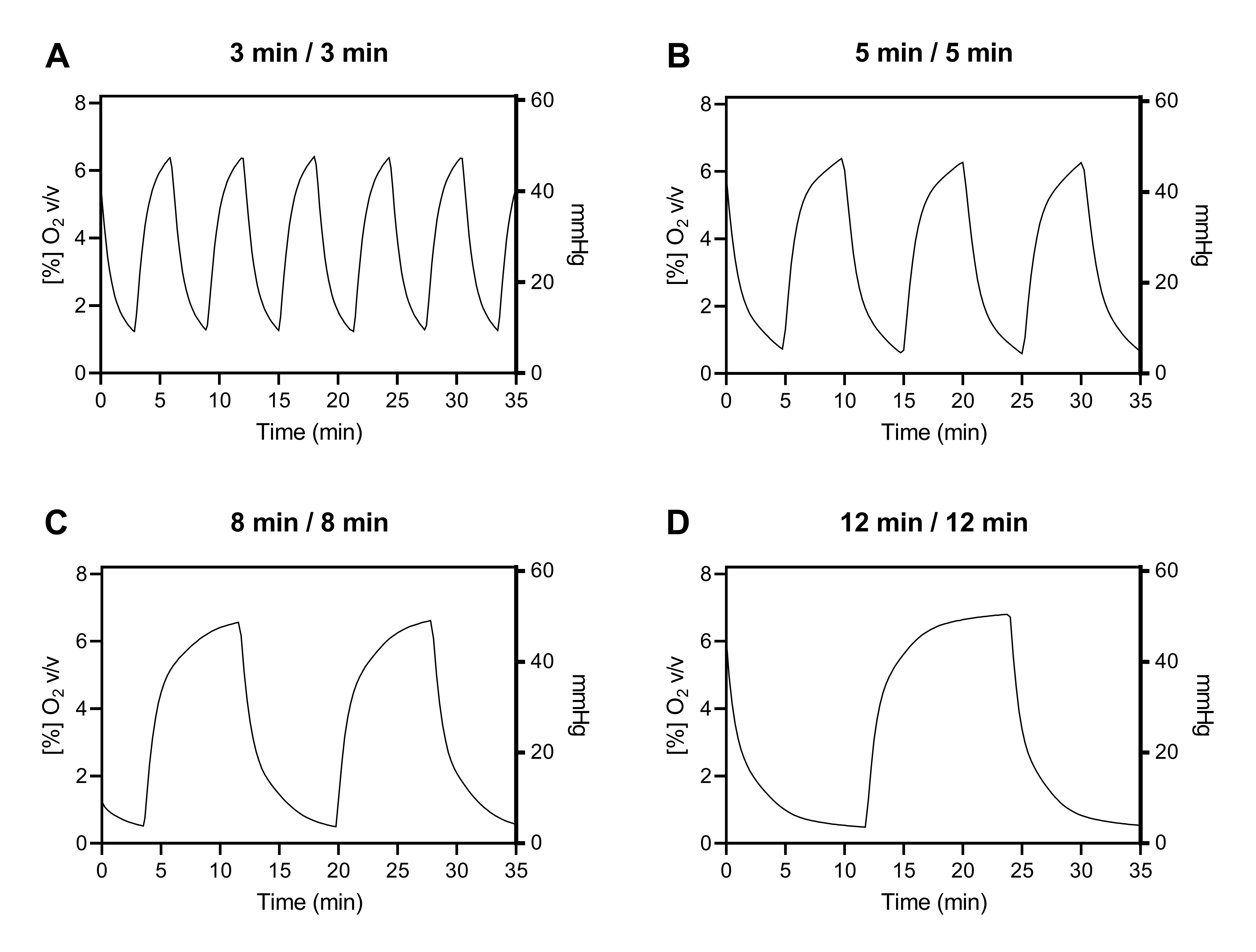


**Supplemental Figure 8.** Oxygen measurements in the peri-cellular media during exposure to intermittent hypoxia with a normoxic phase of 8% O_2_ v/v and a hypoxic phase of 0% O_2_ v/v. (A) 3 min of normoxia followed by 3 min of hypoxia (3 min/3 min), (B) 5 min/5 min, (C) 8 min/8 min, (D) 12 min/12 min. Data from (A) and (B) were previously published in Martinez et al. 2019^38^ and reproduced, with permission from the authors and publisher, Bio-protocol.

**Supplemental Table 1.** Two-way ANOVA of the KDM4 enzyme assay (Figure F and G) comparing KDM4B and KDM4C activity in normoxia vs. chronic hypoxia vs. intermittent hypoxia.

|  | **Quantity** | **Condition** | **95.00% CI of diff.** | **Adjusted P Value** | **Summary** |
| --- | --- | --- | --- | --- | --- |
| Recombinant KDM4B | 100ng | Normoxia vs. Chronic Hypoxia | -0.1324 to 0.1311 | >0.9999 | ns |
|  |  | Normoxia vs. Intermittent Hypoxia | -0.1119 to 0.1516 | 0.9198 | ns |
|  |  | Chronic vs. Intermittent Hypoxia | -0.1113 to 0.1522 | 0.9146 | ns |
|  | 300ng | Normoxia vs. Chronic Hypoxia | 0.1267 to 0.3902 | 0.0004 | *** |
|  |  | Normoxia vs. Intermittent Hypoxia | -0.1297 to 0.1338 | 0.9991 | ns |
|  |  | Chronic vs. Intermittent Hypoxia | -0.3882 to -0.1247 | 0.0004 | *** |
|  | 500ng | Normoxia vs. Chronic Hypoxia | 0.1632 to 0.4267 | <0.0001 | **** |
|  |  | Normoxia vs. Intermittent Hypoxia | -0.2217 to 0.04181 | 0.2120 | ns |
|  |  | Chronic vs. Intermittent Hypoxia | -0.5166 to -0.2531 | <0.0001 | **** |
|  | 800ng | Normoxia vs. Chronic Hypoxia | 0.2983 to 0.5618 | <0.0001 | **** |
|  |  | Normoxia vs. Intermittent Hypoxia | -0.1909 to 0.07253 | 0.4896 | ns |
|  |  | Chronic vs. Intermittent Hypoxia | -0.6210 to -0.3575 | <0.0001 | **** |
| Recombinant KDM4B | 100ng | Normoxia vs. Chronic Hypoxia | -0.1334 to 0.1464 | 0.9920 | ns |
|  |  | Normoxia vs. Intermittent Hypoxia | -0.1150 to 0.1648 | 0.8896 | ns |
|  |  | Chronic vs. Intermittent Hypoxia | -0.1215 to 0.1583 | 0.9380 | ns |
|  | 300ng | Normoxia vs. Chronic Hypoxia | -0.009664 to 0.2702 | 0.0699 | ns |
|  |  | Normoxia vs. Intermittent Hypoxia | -0.2025 to 0.07731 | 0.4925 | ns |
|  |  | Chronic vs. Intermittent Hypoxia | -0.3328 to -0.05295 | 0.0072 | ** |
|  | 500ng | Normoxia vs. Chronic Hypoxia | 0.3714 to 0.6512 | <0.0001 | **** |
|  |  | Normoxia vs. Intermittent Hypoxia | -0.01764 to 0.2622 | 0.0915 | ns |
|  |  | Chronic vs. Intermittent Hypoxia | -0.5290 to -0.2491 | <0.0001 | **** |
|  | 800ng | Normoxia vs. Chronic Hypoxia | 0.3633 to 0.6431 | <0.0001 | **** |
|  |  | Normoxia vs. Intermittent Hypoxia | -0.1188 to 0.1611 | 0.9188 | ns |
|  |  | Chronic vs. Intermittent Hypoxia | -0.6219 to -0.3421 | <0.0001 | **** |

**Supplemental Table 2.** Two-way ANOVA of HCT116 spheroids exposed to normoxia and intermittent hypoxia comparing fluorescence intensity of H3K9me3:Histone H3 in the monolayer vs. periphery vs. core (Figure 4 and Supplemental Figure 7).

|  | **Condition** | **Spheroid layer** | **95.00% CI of diff.** | **Adjusted P Value** | **Summary** |
| --- | --- | --- | --- | --- | --- |
| Plane 1 | Normoxia | Periphery vs. Core | -2.466 to -1.853 | <0.0001 | **** |
|  | Intermittent Hypoxia | Periphery vs. Core | -1.268 to -0.6542 | <0.0001 | **** |
| Plane 2 | Normoxia | Periphery vs. Core | -2.230 to -1.517 | <0.0001 | **** |
|  | Intermittent Hypoxia | Periphery vs. Core | -1.228 to -0.5155 | <0.0001 | **** |
| Plane 3 | Normoxia | Monolayer vs. Periphery | -0.8075 to -0.3205 | <0.0001 | **** |
|  |  | Monolayer vs. Core | -2.384 to -1.897 | <0.0001 | **** |
|  |  | Periphery vs. Core | -1.820 to -1.333 | <0.0001 | **** |
|  | Intermittent Hypoxia | Monolayer vs. Periphery | -0.8791 to -0.3921 | <0.0001 | **** |
|  |  | Monolayer vs. Core | -2.064 to -1.577 | <0.0001 | **** |
|  |  | Periphery vs. Core | -1.428 to -0.9411 | <0.0001 | **** |
